# Prohibitin 1 interacts with p53 in the regulation of mitochondrial dynamics and chemoresistance in gynecologic cancers

**DOI:** 10.1186/s13048-022-00999-x

**Published:** 2022-06-07

**Authors:** Bao Kong, Chae Young Han, Se Ik Kim, David A. Patten, Youngjin Han, Euridice Carmona, Dar-Bin Shieh, Annie C. Cheung, Anne-Marie Mes-Masson, Mary-Ellen Harper, Yong Sang Song, Benjamin K. Tsang

**Affiliations:** 1 Departments of Obstetrics and Gynecology and Cellular and Molecular Medicine, Interdisciplinary School of Health Sciences University of Ottawa, and Chronic Disease Program, Ottawa Hospital Research Institute, 501 Smyth Road, Mail Box #511, ON K1H 8L6 Ottawa, Canada; 2 Department of Obstetrics and Gynecology and Cancer Research Institute, Seoul National University College of Medicine, Seoul, 03080 Republic of Korea; 3 Department of Biochemistry, Microbiology and Immunology, Faculty of Medicine, University of Ottawa, Ottawa, Canada; 4 Centre de recherche du Centre hospitalier de l’Université de Montréal and Institut du cancer de Montréal, Montréal, Canada; 5 Institute of Basic Medical Science, Institute of Oral Medicine and Department of Stomatology, National Cheng Kung University Hospital, College of Medicine, National Cheng Kung University, Tainan, 704 Taiwan; 6 Department of Pathology, The University of Hong Kong, Hong Kong, SAR China

**Keywords:** Phb1, p53, Bak, CDDP, Mitochondrial fragmentation, Chemoresistance

## Abstract

**Background:**

Mitochondrial dynamics (e.g. fission/fusion) play an important role in controlling chemoresistance in representative gynecologic malignancies, ovarian and cervical cancer. Processing the long form of Optic atrophy (L-Opa)1 is a distinctive character of mitochondrial fragmentation, associated with chemosensitivity. Here, we examined the role of prohibitin (Phb)1 in increasing L-Opa1 processing via the regulating mitochondrial protease, Oma1 and its direct interaction with p-p53 (ser15) and pro-apoptotic Bcl-2 antagonist/killer (Bak) 1 in the signaling axis and if this phenomenon is associated with prognosis of patients.

**Methods:**

We compared Cisplatin (CDDP)-induced response of mitochondrial dynamics, molecular interaction among p-p53 (ser15)-Phb1-Bak, and chemoresponsiveness in paired chemosensitive and chemoresistant gynecologic cancer cells (ovarian and cervical cancer cell lines) using western blot, immunoprecipitation, sea horse, and immunofluorescence. Translational strategy with proximity ligation assessment in phb1-p-p53 (ser15) in human ovarian tumor sections further confirmed in vitro finding, associated with clinical outcome.

**Results:**

We report that: (1) Knock-down of Phb1 prevents Cisplatin (*cis*-diamine-dichloroplatinum; CDDP) -induced changes in mitochondrial fragmentation and Oma1 mediated cleavage, and Opa1 processing; (2) In response to CDDP, Phb1 facilitates the p-p53 (ser15)-Phb1-Bak interaction in mitochondria in chemosensitive gynecologic cancer cells but not in chemoresistant cells; (3) Akt overexpression results in suppressed p-p53(Ser15)-Phb1 interaction and dysregulated mitochondrial dynamics, and (4) Consistent with in vitro findings, proximity ligation assessment (PLA) in human ovarian tumor sections demonstrated that p-p53(ser15)-Phb1-Bak interaction in mitochondria is associated with better chemoresponsiveness and clinical outcome of patients. Determining the molecular mechanisms by which Phb1 facilitates mitochondrial fragmentation and interacts with p53 may advance the current understanding of chemoresistance and pathogenesis of gynecologic cancer.

**Conclusion:**

Determining the key molecular mechanisms by which Phb1 facilitates the formation of p-p53 (ser15)-Bak-Phb1 and its involvement in the regulation of mitochondrial dynamics and apoptosis may ultimately contribute to the current understanding of molecular and cellular basis of chemoresistance in this gynecologic cancer.

**Supplementary Information:**

The online version contains supplementary material available at 10.1186/s13048-022-00999-x.

## Introduction

Ovarian cancer (OVCA) and Cervical cancer (CECA) together are the fleading cause of cancer deaths in women [1]. Cisplatin (CDDP: *cis*-diamine-dichloroplatinum) and its analogs (e.g. Carboplatin and Oxaliplatin) are the standard first line chemotherapeutic agents after surgery in the treatment of OVCA patients. However, chemoresistance severely limits treatment success of these two cancer types. Dysregulation of the apoptotic pathway is a mechanism underlying chemoresistance [2]. CDDP-induced, p53-mediated mitochondrial cell death is a determinant of chemosensitivity in gynecologic cancer cells [3–5].

Mitochondria are highly dynamic organelles. Mitochondrial fusion and fission are required for mitochondrial functions and are also involved in the regulation of mitochondria-mediated apoptosis [6, 7]. Mitochondrial dynamics are controlled by a series of proteins, including fusion proteins optic atrophy type (Opa1), mitofusin (Mfn) 1 and 2, and fission proteins dynamin-related protein 1 (Drp1) and Mitochondrial fission 1 protein (Fis1) [6, 8]. Opa1 regulates mitochondrial structure and function through the proper assembly of its oligomerization, which requires the optimal balance of inner membrane bound long and short soluble Opa1 isoforms [9, 10]. The latter are the products of proteolytic processing by two metalloproteases: the membrane potential-dependent protease Oma1 and the ATP-dependent protease Yme1L [11]. This proteolytic cleavage regulates Opa1 function [12].

Oma1-mediated processing of Long-form of Opa1 (L-Opa1) and disruption of Opa1 oligomers are required for mitochondrial fragmentation, pro-apoptotic cytochrome c release and subsequent cell death [12, 13]. Our previous report has shown that CDDP induces mitochondria fragmentation in chemosensitive CECA and OVCA cells, but not in chemoresistant counterparts. Oma1-mediated L-Opa1 processing is different in chemosensitive and chemoresistant cells in response to CDDP treatment [14, 15], suggesting that mitochondrial dynamics may play an important role in regulating chemoresistance. We also found that p53 is required for Oma1-mediated L-Opa1 processing, although the mechanism involved requires further investigation [14].

Prohibitins (Phb) form large ring complexes in the mitochondrial inner membrane containing Phb1 and Phb2 subunits, composed of N terminal transmembrane units that function as membrane scaffolds [16]. Phb1 and Phb2 are interdependent since the loss of one simultaneously leads to the loss of the other [17]. Phb1 is a shuttle protein and newly synthesized Phb1 and Phb2 in mitochondria need to pass through the nuclear pore complex and translocates to the nucleus for transcriptional regulation [18].

Phb1, a multifunctional protein, has been implicated in different cellular processes, including the regulation of cell cycle progression, apoptosis and gene transcription [17, 19, 20]. L-Opa1 processing and mitochondrial dynamics are regulated by Phb1 [17, 19]. p53 was shown to interact with Phb1 during apoptotic signaling and that the function of p53 is attenuated in the absence of Phb1, suggesting that Phb1 plays an important role in p53-regulated apoptosis [21, 22]. The role of Phb1 has been shown to be both pro-survival or pro-apoptotic and appears to be cancer type-specific [23]. However, precisely how Phb1 regulates mitochondrial dynamics in cervical and ovarian cancers has not been fully investigated.

BCL2-antagonist/killer (Bak) is a pro-apoptotic Bcl-2 family member that exists as a globular monomeric protein in the mitochondria. Apoptotic stimuli induce the exposure of the Bcl-2 homology domain 3 (BH3), which binds to the hydrophobic surface groove of another member, resulting in the formation of Bak homodimers. These homodimers further assemble into pore-forming oligomers, contributing to mitochondrial outer membrane permeabilization (MOMP) [24]. Interestingly, p-p53 (ser15) has been reported to interact with Bak, leading to spontaneous oligomerization of Bak and MOMP [25]. Whether Phb1 is involved in the interaction of p-p53 (ser15) and Bak, and how this is related to Oma1-mediated L-Opa1 processing and mitochondrial fragmentation, need to be investigated. In addition, Akt is an important cell survival factor and is known to be activated or overexpressed in different cancer types [26]. We have previously demonstrated that Akt promotes chemoresistance through inhibition of p53 phosphorylation and its action on caspase-dependent mitochondrial death pathway [3, 4]. However, the mechanism through which p53 elicit its action is not clear.

Here, we examine the role of p-p53 (ser15) in mitochondrial dynamics and apoptosis, its Akt-dependent mitochondrial translocation, and subsequent Phb1 and Bak interactions in apoptotic signaling. We have observed that p-p53(ser15) interacts with Bak during mitochondrial fragmentation and apoptosis, a process involving the participation of Phb1. Furthermore, this pathway is modulated by Akt in chemoresistant cells.

## Materials and methods

### Reagents

CDDP, DMSO, Hoechst 33258, phenylmethylsulfonyl fluoride (PMSF), sodium orthovanadate (Na_3_VO_4_), and aprotinin were purchased from Sigma-Aldrich (St Louis, MO, USA). Antibodies used in the present study are described in Supplementary Table S1. Phb1 siRNA and scramble siRNA were purchased from Origene (Rockville, MD, USA). HA-tagged, triple-A mutated (K179A, T308A, S473A) DN-Akt (dead kinase)- and LacZ adenoviral constructs were synthesized at the University of Ottawa Adenoviral Core Facility (Ottawa, ON, Canada).

### Cell lines and cell culture

The CDDP-sensitive cancer cell OV2008 (wt-p53, mutant-PI3K) is of cervical origin. CDDP-resistant C13* (wt-p53, mutant-PI3K) cell line is the isogenic resistant counterpart to OV2008, selected by chronic exposure to increasing concentrations of CDDP in vitro. CDDP-sensitive A2780s (wt-p53) and its resistant variant A2780cp (mutant-p53) are endometrioid sub-type of epithelial ovarian cancer [4, 27]. Detailed information of cell lines are described in Supplementary Table S2. These cell lines were gifts from Drs. Rakesh Goel and Barbara Vanderhyden (Ottawa Regional Cancer Centre, Ottawa, Ontario, Canada), and were cultured as previously reported [3, 4].

### Clinical ovarian tumor sections

Under IRB-approved protocols at the Ottawa Health Science Network Research Ethics Board (OHSN-REB Protocol No. 20150646-01H) and collaborating institutions, including the Seoul National University Hospital (IRB No. H-1711-142-904), University of Hong Kong (IRB UW16_107), and CRCHUM (IEC No. 2005–1893, BD 04.002 – BSP), formalin-fixed-paraffin-embedded (FFPE) ovarian tumor sections were collected and assessed. The stage, histology, and tumor grades were determined using the criteria of the International Federation of Gynecology and Obstetrics (FIGO) classification. Pre-chemotherapy and post-chemotherapy ovarian tumor sections were obtained at primary and secondary cytoreductive surgery, respectively except for 2 neoadjuvant cases. Additional patient data are provided in Supplementary Table S3.

### Protein extraction and Western blot analysis

Protein extraction and Western blot analysis were performed as previously described [14, 28]. Unless indicated otherwise, membranes were incubated overnight at 4 °C with diluted antibodies (Supplementary Table S1) and band densities were analyzed (Scion Image software; Scion Corporation, Frederick, MD, USA).

### Fluorescence microscopy and determination of mitochondrial phenotype

These procedures were performed as previously described [14, 28]. Cells were plated on poly-D-lysine-coated (0.05% w/v; Sigma) 8-well glass culture slides (BD Biosciences) and cultured (48 h) in RPMI 1640 (growth medium) prior to CDDP treatment. For immunostaining, cells were fixed in paraformaldehyde (4%, 1 h, RT), washed in PBS, and blocked with 1% BSA. Mitochondria were visualized by immunofluorescence microscopy, using a mouse monoclonal antibody anti-human Tom 20 (1:100; Santa Cruz Biotechnology) and Alexa Fluor 488 goat anti-mouse secondary antibody (1:500; Invitrogen). Confocal images were obtained (× 100 objective) on an Olympus IX81 inverted microscope with appropriate argon lasers (488 nm). Mitochondrial phenotype of each cell was categorized as being tubular, intermediate or fragmented, as previously described [14]. At least 100 cells were analyzed per treatment group.

### RNA interference

For gene knock-down studies, cells were transfected with Phb1 siRNA (0–100 nM; 24 h), or control siRNA (scrambled sequence), and were treated with CDDP (0–10 μM; 24 h) as previously described [14], and harvested for further analysis.

### Assessment of apoptosis

Apoptosis was assessed morphologically by Hoechst 33258 dye (6.25 ng/ml). At least 400 cells/treatment groups were counted. Selected fields and blinded slides were determined randomly to avoid experimental bias [28].

### Adenoviral infection

C13* cells were infected with adenoviral HA-DN-Akt (MOI = 0–80, 24 h), as previously described [4]. Adenoviral LacZ served as a control.

### Cellular fractionation

Mitochondrial, nuclear and cytosolic fractions were prepared as previously described [29]. Briefly, cells were washed with ice-cold phosphate buffered saline at the end of the culture period, left on ice for 10 min, and then resuspended in homogenizing buffer containing the protease inhibitors (Aprotinin, Na_3_VO_4_ and PMSF). After 60 strokes in a Dounce homogenizer, the unbroken cells were spun down (30 g; 5 min). The nuclear and heavy mitochondrial fractions were collected at 750 g (10 min) and 14,000 g (20 min), respectively, and the resulting supernatant was kept as cytoplasm. The nuclear fraction was washed three times with the homogenizing buffer containing 0.01% NP-40.

### Immunoprecipitation

One mg of protein sample was incubated (RT, 1 h) with 50 μL Protein G Dynabeads (Invitrogen) coated with rabbit monoclonal Bak antibody (1 μg, Abcam) and immunoprecipitated. The beads were pelleted, and re-suspended in sample buffer, boiled, and loaded onto 9% SDS-PAGE. After protein transferred to nitrocellulose, Phb1 and p-p53 (ser15) contents were examined by Western blotting, using the Clean Blot IP Detection Reagent (Thermo Fisher Scientific, Waltham, MA, USA).

### Extracellular flux assays

Mitochondrial stress test was performed for the measurements of the oxygen consumption rate (OCR) using the Seahorse XF96e Extracellular Flux Analyzer (Agilent, Santa Clara, CA, USA). A2780s and A2780cp cells were evenly seeded with a density of 20,000 cells/well (experimentally determined) on XF96e cell culture microplate a day before experiments. Prior to the experiment, culture medium was replaced with XF96e DMEM and incubated in a non-CO_2_ incubator (37 °C, 1 h). Oligomycin A (1 μM; for ATP synthase inhibition), FCCP (0.5 μM; for maximal respiratory capacity), antimycin A [(0.5 μM)/ rotenone (1 μM) for terminating mitochondrial respiration] were sequentially added into each well for the assessment of resting OCR, ATP-linked OCR, and maximal respiratory capacity, respectively. OCR was measured over a 3 min- period and each values were normalized to the protein concentration in each well, as determined by the Bradford assay.

### Proximity ligation assay (PLA)

PLA was conducted using the Duolink Detection kit (Sigma, St. Louis, MO, USA), as previously described [30, 31] and as per the manufacturer’s instructions. For cultured cells, cells (30,000–40,000 cells per well of 8 chamber slide) were cultured and treated as indicated. Cells were fixed and incubated overnight (16 h) with a pair of anti-P-p53(ser15) and anti-Phb1 antibodies as indicated in supplementary section (Supplementary Table S1), followed by secondary proximity probes (Duolink In Situ PLA probe anti-rabbit plus, DUO92002 and anti-mouse minus, DUO92004). Then, samples were incubated with the Detection Reagents Orange (DUO92007) containing T4 DNA ligase (1 unit/μl) and amplification solution (DNA polymerase, 10 units/μl) with a fluorophore (554 nm excitation and 676 nm emission) followed by counterstaining with TOM 20 (mitochondrial marker) or DAPI (nucleus marker). Fluorescence signals were detected (64X objective) by confocal microscopy (LSM 510, ZEISS). PLA-positive signals were quantified using Duolink Image Tool (Sigma-Aldrich). At least 50–100 cells were analyzed per experimental group. Detailed quantification method and untreated representative control cells were shown in Supplementary Fig. 1.

For ovarian tumors, 4–5 μm sections were cut. Three to five non-necrotic fields with 1 mm^2^ areas are randomly selected across tumors. Sections were heated in citrate buffer for antigen-retrieval (25 min) and incubated with anti-p-p53(Ser15) and HK2 antibodies. All subsequent PLA steps are the same as mentioned above in (A). Cut off value of low and high PLA expression [Phb1-p-p53(ser15)] was assigned a median value of score < 0.68, and > 0.68, respectively. Kaplan-Meier curves were stratified with log-rank method accordingly.

### Statistical analysis

Results are expressed as the means ± s. e. m. of at least three independent experiments. Statistical analysis was carried out by two-way ANOVA, using the PRISM software (Version 6.0; GraphPad, San Diego, CA, USA). Differences between multiple experimental groups were determined by the Bonferroni post-hoc test. Statistical significance was inferred at *p* < 0.05.

## Results

### CDDP increases Phb1 content, mitochondrial fragmentation and apoptosis in chemosensitive but not chemoresistant OVCA/CECA cancer cells

**Fig. 1:**
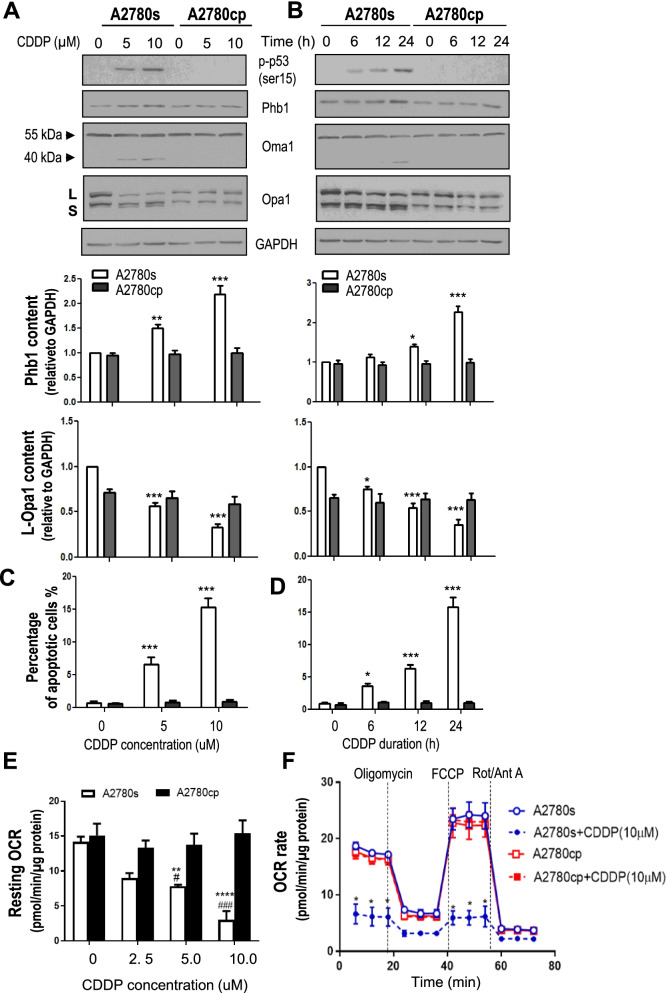
CDDP induces changes in mitochondrial fragmentation, apoptosis and oxygen consumption in A2780s cells, but not in A2780cp cells. A2780s and A2780cp cells were cultured with CDDP at **(A)** different concentrations (0–10 μM, 24 h) or for **(B)** different duration (0–24 h, 10 μM). Protein contents of Long form (L) and short form (S) of Opa1, Oma1, p-p53 (ser15), Phb1, and GAPDH (loading control) were examined by Western blotting (WB). **C** and **D** Morphological assessment of apoptosis was examined by Hoechst nuclear assay in cells cultured above in **(C)** concentration- and **(D)** culture duration- dependent manner. **E** For measurement of mitochondrial respiration, A2780s and A2780cp cells were seeded on 96-well plate, treated with CDDP and resting oxygen consumption rate (OCR) were measured after exposure to biomodulators as indicated (dashed vertical line), using an XF96e Extracellular Flux Analyzer. **F** OCR measurements were obtained over time (72 min) in A2780s and A2780cp cells cultured in the absence (0 μM) and maximal concentration of CDDP (10 μM). For all experiments described, DMSO was used as vehicle control. Results are expressed as mean ± SEM (*n* = 3) and analyzed by 2-way ANOVA and Bonferroni post-hoc test. [*p < 0.05, **p < 0.01, ***p < 0.001 (versus absence of CDDP), *n* = 3]

Although Phb1 has been reported to be involved in the regulation of L-Opa1 processing and mitochondrial dynamics [17, 19, 20], whether it plays a role in CDDP-induced mitochondrial fragmentation and apoptosis in OVCA cells is not known. Chemosensitive A2780s (p53 wt) and its chemoresistant counterpart A2780cp (mutant-p53) OVCA cells were cultured with CDDP (0–10 μM, 0–24 h) and the changes in Phb1 protein contents were examined by Western blot. Phb1 content significantly increased in a concentration- (5 and 10 μM CDDP, 24 h; Fig. 1A *p* < 0.01 *p* < 0.001) and time-dependent manner (0-24 h, 10 μM CDDP; Fig. 1B in response to CDDP in A2780s cells but not in A2780cp cells (*p* < 0.05, *p* < 0.001, Fig. 1A, B). We also observed markedly increased protein content of p-p53 (ser15), cleaved Oma1 (40 KDa) and Phb1. In addition, CDDP decreased L-Opa1 in a concentration- (*p* < 0.001,) and time- (*p* < 0.05, *p* < 0.001, Fig. 1A, B) dependent manner in A2780s cells but not in A2780cp cells. This response was also associated with increased CDDP-induced apoptosis (as determined morphologically by Hoechst staining) in chemosensitive A2780s cells but not in A2780cp cells (*p* < 0.05, *p* < 0.001, Fig. 1 A, B). CDDP induced apoptosis in A2780s cells, but not in A2780cp cells (*p* < 0.05, Fig. 1C, D). We also extended the above studies to include the chemosensitive OV2008 (p53-wt) and chemoresistant C13* CECA cells (mutant-p53). Consistently, we observed increased Phb1 protein content, associated with increased apoptosis in chemosensitive but not in chemoresistant cells (Supplementary Fig. 2A, 2B).

Following the observed changes in the above key mitochondrial structure proteins, we then examined mitochondrial bioenergetics, using the Seahorse XF96e Analyzer, and determined the oxygen consumption rate (OCR),an indicator of oxidative phosphorylation. As shown in Fig. 1E, CDDP markedly decreased the levels of resting OCR in chemosensitive A2780s cells, but not in resistant A2780cp cells (*p* < 0.01, Fig. 1E). In addition, CDDP treatment resulted in a significant decrease in both resting and maximal OCR rate over time in chemosensitive cells (*p* < 0.01, Fig. 1F), suggesting that key function of mitochondria and its bioenergetics were affected by CDDP in chemosensitive OVCA cells but not in chemoresistant cells.

### Phb1 is required for CDDP-induced Oma1-mediated L-Opa1 processing, mitochondrial fragmentation, and apoptosis

**Fig. 2:**
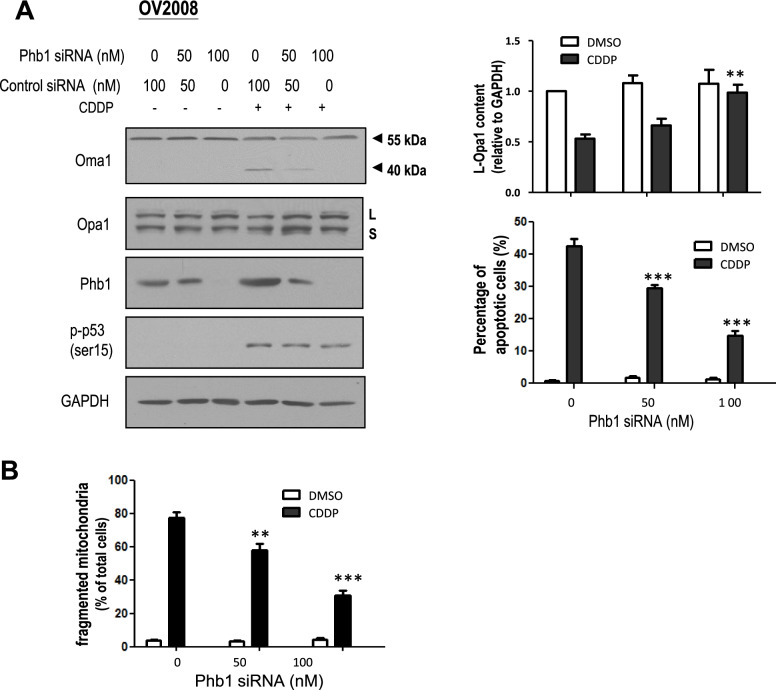
CDDP-induced, Phb1-dependent Oma1 cleavage, L-Opa1 processing, mitochondrial fragmentation and apoptosis in chemosensitive CECA cells. **A** OV2008 cells were treated with Phb1 siRNA (0–100 nM, 24 h; scrambled siRNA as control), and subsequently treated with CDDP (0–10 μM, 24 h). The contents of Oma1, Phb1, Opa1, p-p53 (ser15), and GAPDH (loading control) were determined by WB (Left upper panel). Apoptosis was examined by Hoechst assay (Right bottom panel). **B** OV2008 cells were treated with control siRNA or Phb1 siRNA (0–100 nM, 24 h), followed by CDDP (0–10 μM, 6 h). Mitochondrial phenotypes were examined by immunofluorescence confocal microscopy. Knock-down of Phb1 inhibited CDDP-induced complete mitochondrial fragmentation. Results are expressed as mean ± SEM (*n* = 3) and analyzed by 2-way ANOVA and Bonferroni post-hoc test. [*p < 0.05, **p < 0.01, ***p < 0.001 (versus CDDP group in absence of Phb1 siRNA); *n* = 3]

Based on our previous finding that Phb1 is involved in CDDP-induced p53 activity, and Phb1 was increased in chemosensitive OVCA and CECA with CDDP treatment [14], we hypothesized that Phb1 is required for Oma1-mediated L-Opa1 processing and apoptosis. To test this hypothesis, OV2008 cells were treated with Phb1 siRNA (0–100 nM, 24 h; scramble siRNA as control) followed by CDDP (0–10 μM, 24 h). The contents of Oma1, Opa1, Phb1, p-p53 (ser15) and GAPDH (loading control) were determined by Western blot. Phb1 knock-down significantly stabilized Oma1 (40 KDa) and inhibited its cleavage of L-Opa1 in response to CDDP (Fig. 2A, *p* < 0.01) and apoptosis (Fig. 2A, *p* < 0.001). Notably, the increase of p-p53 (ser15) induced by CDDP was not affected by Phb1 knock-down (Fig. 2A). Knock-down of Phb1 inhibited CDDP-induced mitochondrial fragmentation (Fig. 2B, *p* < 0.01, *p* < 0.001). Taken together, these data suggest that Phb1 is required for CDDP-induced Oma1 (40 KDa) increase, L-Opa1 processing, associated with mitochondrial fragmentation.

### Chemoresponsiveness is associated with increased p-p53 (ser15)-Phb1-Bak interaction in CECA cells

**Fig. 3:**
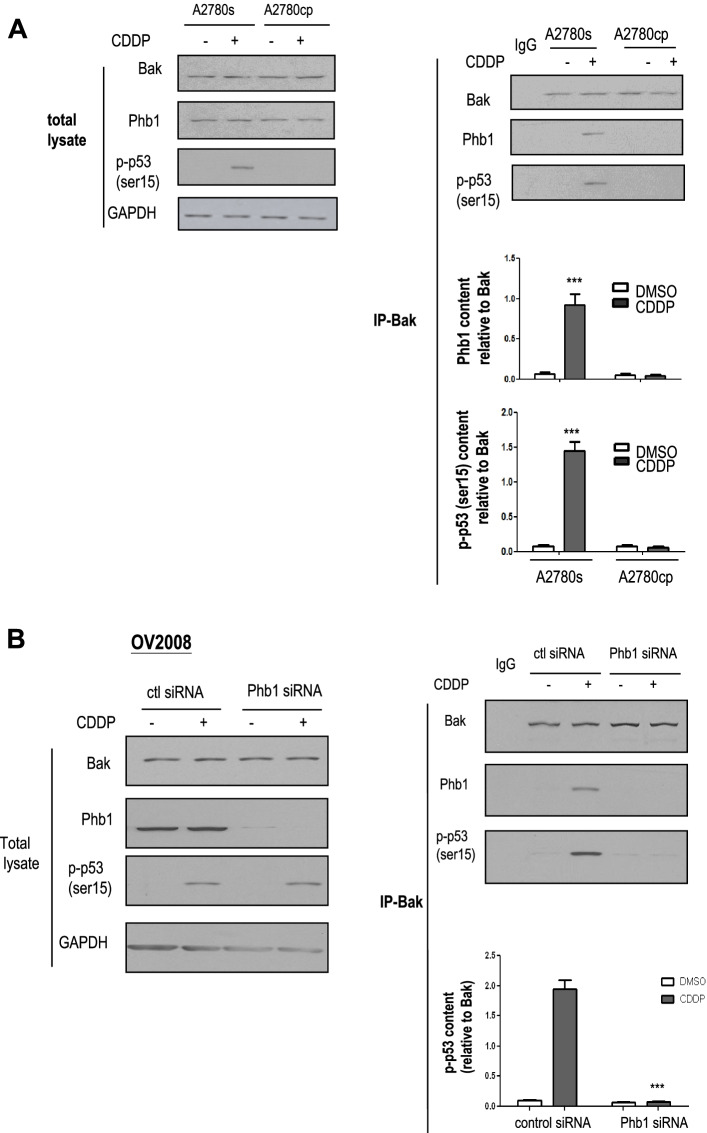
CDDP induces p-p53 (ser15)-Phb1-Bak interaction in chemosensitive but not in chemoresistant gynecologic cancer cells. **A** A2780s (chemosensitive OVCA cells) and A2780cp (chemoresistant OVCA cells) were treated with CDDP (0–10 μM, 6 h). Protein contents of Phb1, p-p53 (ser15), Bak, and GAPDH were examined by WB. Protein-protein interaction was determined by immunoprecipitation (IP)-WB. Cell lysates were immunoprecipitated with anti-IgG (control; lanes 1) or anti-Bak antibody. Bak immunoprecipitates were immunoblotted [IP: anti-Bak, WB: anti-Bak, −Phb1, −p-p53 (ser15)]. **B** CDDP-induced Phb1-dependent p-p53 (ser15)-Phb1-Bak interaction. OV2008 (chemosensitive CECA cells) were forced expressed with Phb1 siRNA (0–100 nM, 24 h; scrambled siRNA as control) and then treated with CDDP (0–10 μM, 6 h). Contents of Phb1, p-p53 (ser15), Bak and GAPDH were examined by WB. Cell lysates were immunoprecipitated with IgG (control; lanes 1) or Bak antibody. Protein-protein interaction was determined by IP-Western. Bak immunoprecipitates were immunoblotted [IP: anti-Bak, WB: anti-Bak, −Phb1, −p-p53 (ser15)]. Results are expressed as mean ± SEM (*n* = 3) and analyzed by 2-way ANOVA and Bonferroni post-hoc test. [*p < 0.05, **p < 0.01, ***p < 0.001 (versus DMSO **(A)** and CDDP in absence of Phb1siRNA **(B)**); *n* = 3]

We have previously found that p-p53 (ser15) interacts with Phb1 in response to CDDP treatment in OV2008 cells [14]. Additionally, others have demonstrated that p-p53 (ser15) accumulates in mitochondria, and binds to Bak, leading to spontaneous Bak oligomerization and changes in MOMP [25]. Based on these findings, we postulate that p-p53 (ser15) binds to Bak in response to CDDP, and this is associated with mitochondrial-mediated apoptosis. To investigate this hypothesis, A2780s and A2780cp cells were treated with CDDP (0–10 μM, 6 h) and Bak immunoprecipitates were immunoblotted with Bak, Phb1, and p-p53 (ser 15). CDDP induced the interaction of p-p53 (ser 15) and Bak in A2780s cells (*p* < 0.001), but not in A2780cp cells (Fig. 3A). CDDP also increased Phb1-Bak interaction in A2780s cells (*p* < 0.001), but not in A2780cp cells, suggesting that Phb1 may be required for Bak oligomerization (Fig. 3A).

In addition, we observed a similar phenomenon in CECA cells; CDDP increased p-p53 protein content at both ser15 and ser20 sites. CDDP also increased p-p53 (ser 15) - Bak interaction in chemosensitive OV2008 cells (*p* < 0.001), but not in chemoresistant C13* cells (Supplementary Fig. 3). However, CDDP failed to increase interaction of p-p53 (ser20) and Bak and this interaction was not detectable in Bak immunoprecipitates from both OV2008 and C13* cells, regardless of CDDP treatment (Supplementary Fig. 3). We further demonstrated that Phb1 is involved in CDDP-induced Bak-p-p53 (ser15) interaction in OV2008 cells with IP/WB (Fig. 3A). In addition, Phb1 knock-down inhibited CDDP-induced interaction of Bak with p-p53 (ser15) (*p* < 0.001, Fig. 3B).

### Akt confers resistance by suppressing interaction of p-p53 (ser15), Phb1 and Bak and key proteins involved in mitochondrial fragmentation

**Fig. 4:**
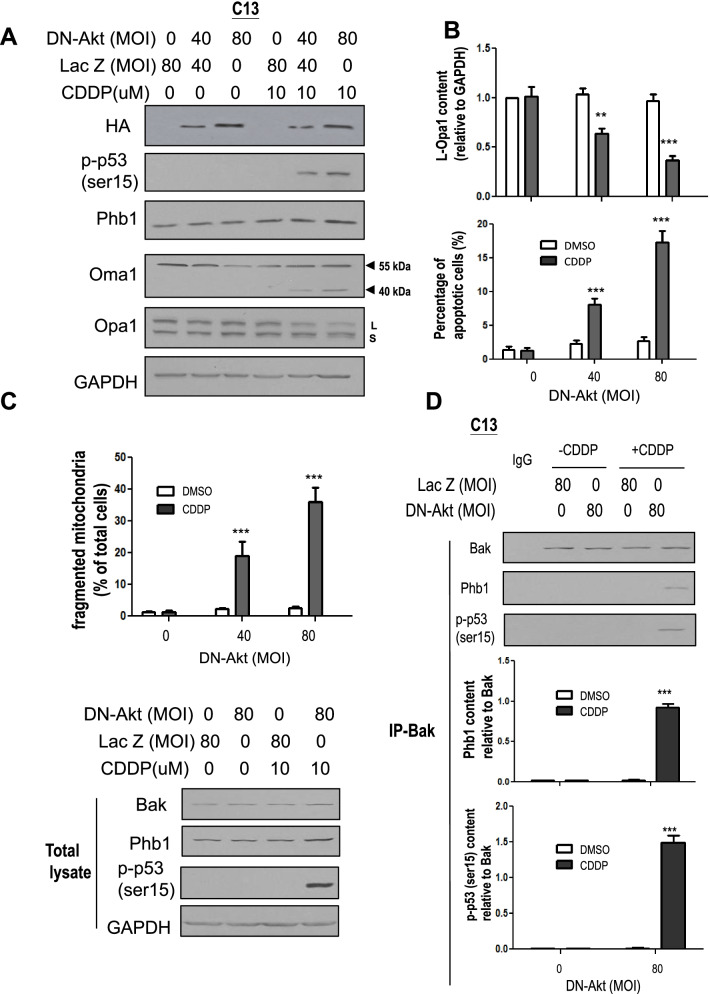
Akt confers CDDP resistance by suppressing Oma1 cleavage, L-Opa1 processing, mitochondrial fragmentation and apoptosis. **A** HA-DN-Akt treatment enhanced CDDP-induced p-p53 (ser15), Oma1 (40 KDa) and L-Opa1 processing [**p < 0.01, ***p < 0.001, (versus CDDP in absence of DN-Akt); *n* = 3]. C13* (chemoresistant CECA cells) were forced expressed with HA-DN-Akt or Lac Z (Multiplicity of Infection (MOI) = 0–80, 24 h) and treated with CDDP (0–10 μM, 24 h). The contents of HA tag, p-p53 (ser15), Phb1,Oma1, Opa1, and GAPDH (loading control) were determined by WB. **(B)** HA-DN-Akt treatment sensitized C13 cells to CDDP-induced apoptosis [***p < 0.001 (versus CDDP in absence of DN-Akt); *n* = 3]. C13* cells were treated with HA-DN-Akt or Lac Z (MOI = 0–80, 24 h), and then with CDDP (0–10 μM, 6 h). Apoptosis was examined by Hoechst assay. **C** Akt down-regulation sensitized C13 cells to CDDP-induced mitochondrial fragmentation [***p < 0.001 (versus CDDP in absence of DN-Akt); *n* = 3]. Mitochondrial phenotypes were examined by immunofluorescence confocal microscopy. **D** Akt conferred resistance in C13 cells by suppressing CDDP-induced the interaction of p-p53 (ser15) with Phb1 and Bak. C13* cells were treated with HA-DN-Akt or Lac Z (MOI = 0 or 80, 24 h), and then with CDDP (0–10 μM, 6 h). Contents of Bak, Phb1, p-p53 (ser15), and GAPDH were examined by WB. Cell lysates were immunoprecipitated with IgG (control; lanes 1) or Bak antibody. Protein-protein interaction was determined by IP-Western. Bak immunoprecipitates were immunoblotted [IP: anti-Bak, WB: anti-Bak, Phb1, p-p53 (ser15)]. Results are expressed as mean ± SEM (*n* = 3) and analyzed by 2-way ANOVA and Bonferroni post-hoc test. [***p < 0.001 versus CDDP in absence of DN-Akt); *n* = 3]

Our previous study has shown that Akt confers chemoresistance by suppressing CDDP-induced p53 phosphorylation [4]. We therefore hypothesized that Akt attenuates p-p53 (ser15) phosphorylation, suppresses Oma1 activation, subsequent L-Opa1 processing and mitochondrial fragmentation. To test these possibilities, chemoresistant C13* cells were transfected with HA-tagged dominant negative Akt (HA-DN-Akt, MOI = 0–80, 24 h), and then treated with CDDP (0–10 μM, 24 h). DN Akt with its triple mutation site of K179A, T308A, and S473, including kinase binding site of substrate which interfers with the function of Akt phosphorylation and p53 suppression, as we previously showed [32]. Successful infection was confirmed by Western blot (anti-HA), as were the protein contents of Oma1, Phb1, Opa1, p-p53 (ser15) and GAPDH (loading control). As expected, DN-Akt expression enhanced CDDP-induced p-p53 (ser15) and Oma1 (40 KDa) contents and L-Opa1 processing (*p* < 0.001, Fig. 4A). HA-DN-Akt also significantly sensitized C13* to CDDP-induced apoptosis (*p* < 0.001, Fig. 4B) and mitochondrial fragmentation (*p* < 0.001, Fig. 4C).

We also examined the role of Akt in regulating the interaction between Phb1, Bak and p-p53 (ser15), and mitochondrial fragmentation. HA-tagged DN-Akt were forcibly expressed in C13* cells (0–80 MOI, 24 h) followed by CDDP treatment (0–10 μM, 6 h). DN-Akt expression notably enhanced CDDP-induced interaction of Phb1 with Bak and p-p53 (ser15) (Fig. 4D), suggesting that Akt takes a critical role in suppressing p-p53 (ser15)-Phb1-Bak interaction.

### CDDP induces mitochondrial p-p53 (ser15) accumulation and binding to Phb1 and Bak

**Fig. 5:**
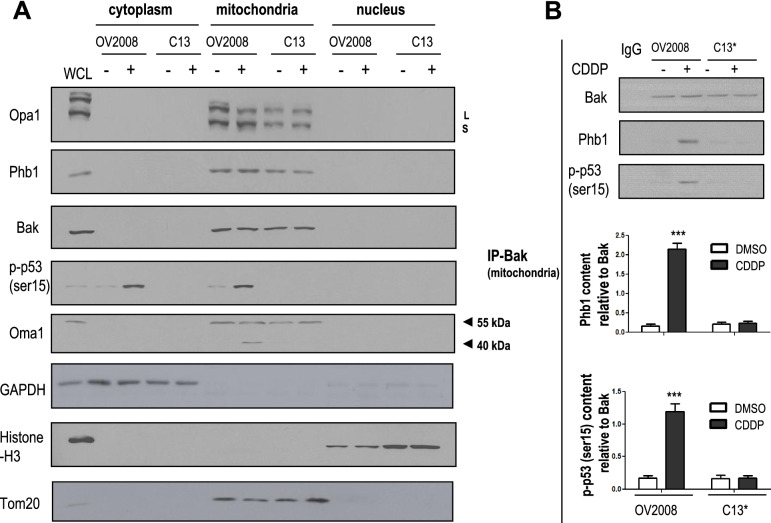
CDDP induced mitochondrial p-p53 (ser15) accumulation and binding to Phb1 and Bak in CECA cells. **A** OV2008 (chemosensitive) and C13* (chemoresistant) cells were treated with CDDP (0–10 μM, 6 h). Mitochondrial, cytoplasmic and nuclear fractions were prepared. Contents of Opa1, Oma1, Phb1, p-p53 (ser15), Bak, Histone H3 (nuclear loading control), Tom20 (mitochondria loading control), and GAPDH (cytoplasm loading control) in the above subcellular fractions were examined by WB. **B** OV2008 and C13* cells were treated with CDDP (0–10 μM, 6 h). Mitochondria fraction was separated and immunoprecipitated with anti-IgG (control; lanes 1) or anti-Bak antibody. Protein-protein interaction was determined by IP-Western. Bak immunoprecipitates were immunoblotted [IP: anti-Bak, WB: anti-Bak, −Phb1 and -p-p53 (ser15)]. Results are expressed as mean ± SEM (*n* = 3) and analyzed by 2-way ANOVA and Bonferroni post-hoc test. [***p < 0.001(versus OV2008 treated with DMSO), *n* = 3]

Our finding and those of others indicate that phosphorylation of p53 and mitochondrial p-p53 accumulation is important for chemoresponsiveness in gynecologic cancer cells [3, 4]. We next investigated whether CDDP induces mitochondrial p-p53 (ser15) accumulation and Phb1-Bak interaction, and activates Oma1-mediated L-Opa1 processing. OV2008 and C13* CECA cells were cultured with CDDP (0–10 μM, 6 h). Using cellular fractions, we assessed the subcellular localization of Opa1, Oma1, Bak, Phb1 and p-p53 (ser15). As expected, Opa1, Oma1, Bak and Phb1 were primarily located in mitochondria in OV2008 and C13* cells, irrespective of the presence of CDDP. Whereas long form of Oma1 (55 KDa) was present in mitochondria fraction in both OV2008 and C13* cells and not affected by CDDP treatment, CDDP increased the content of short form of Oma1 (40 KDa) in chemosensitive OV2008 cells, but not in chemoresistant C13* cells (Fig. 5A). CDDP increased p-p53 (ser15) in cytoplasm and mitochondria in OV2008 cells, but not in C13* cells (Fig. 5A). Qureshi et al. and others previously reported other cellular localization for Phb1, including nucleus and membrane in addition to mitochondria [33, 34]. Interestingy, we found that prolonged CDDP culture (6-24 h) induced mitochondrial localization of Phb1 in both OV2008 and C13* cells whereas Phb1 was still present in the nucleus in absence or shorter treatment of CDDP (0-3 hr) in these cells (Supplmentary Fig. 4).

The interaction of Phb1, Bak and p-p53 (ser15) was further confirmed by IP-Bak in mitochondria fraction. CDDP increased Phb1-Bak and p-p53 (ser15)-Bak interactions in OV2008 cells (*p* < 0.001), but not in C13* cells (Fig. 5B). These findings indicate that the interaction of Phb1-Bak and p-p53 (ser15)-Bak occurs in mitochondria, and is associated with its chemoresponsiveness.

### Mitochondrial Phb1-p-p53(Ser15) interaction is associated with chemosensitivity in OVCA cells

**Fig. 6:**
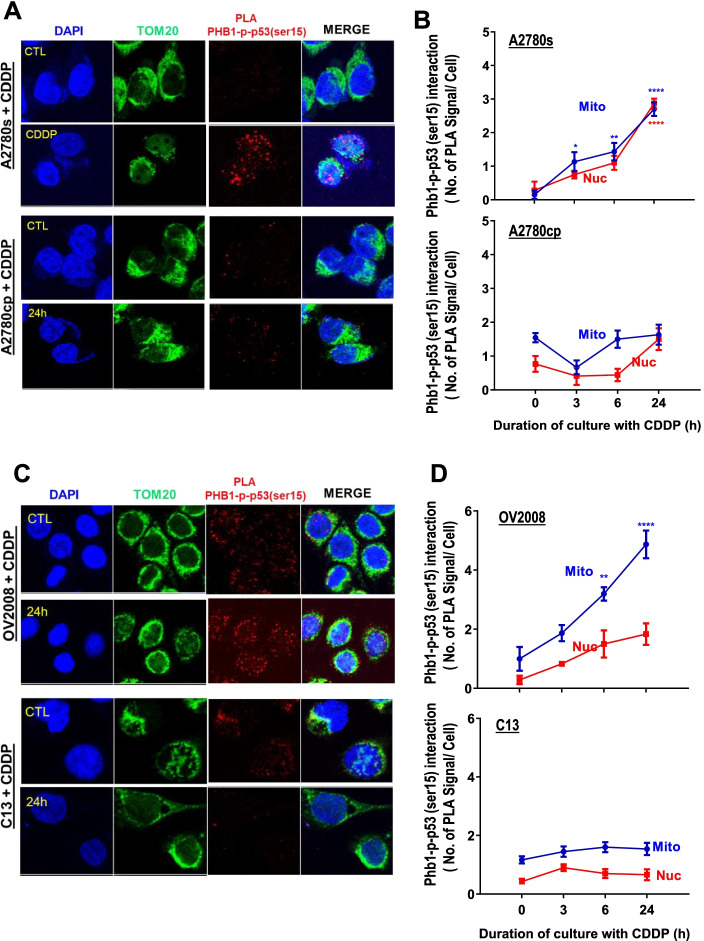
CDDP promotes the interaction of Phb1 and p-p53 (Ser 15) in mitochondria in chemosensitive but not chemoresistant OVCA and CECA cells. Examination of mitochondrial interaction of Phb1-p-p53 (ser15) PLA signal in OVCA and CECA cells lines. A2780s and A2780cp OVCA cells **A** as well as OV2008 and C13 CECA cells (**C)** were cultured with CDDP (10 μM, 0–24 h). and DMSO is used as a vehicle. Interaction of Phb1 and p-p53(ser15) in the mitochondria and nucleus were assessed by using Duolink Image Tool [Number of PLA unit (A: red spot) with counter staining of DAPI (Blue), and TOM 20 (Green)], **(B, D** respectively***)***. Results are expressed as mean ± SEM (*n* = 3) and analyzed by 2-way ANOVA and Bonferroni post-hoc test. [****p < 0.0001 (versus time = 0 h with CDDP); *n* = 3]

Our previous in vitro findings (Fig. 3A) indicate that Phb1 is required for the interaction between p-p53 (ser15) and Bak, a process associated with mitochondrial-mediated apoptosis. Proximity ligation assay (PLA) enables us to determine the cellular localization of Phb-1-P-p53(ser15) interaction in response to CDDP at different time points. Consistent with our in vitro IP results (Fig. 3), CDDP treatment (10 μM, 0–24 h) significantly promoted mitochondrial interaction of Phb1 and p-p53 (ser15) starting at 3 h and reached at maximum of 24 h (Fig. 6A, B, *p* < 0.001) in chemosensitive A2780s, but not in its chemoresistant counterpart A2780cp cells.

Similarly, in chemosensitive CECA cells (OV2008) treated with CDDP (10 μM, 0–24 h), mitochondrial Phb1-p-p53 (ser15) interaction was increased starting at 6 h and reached the highest levels at 24 h (Fig. 6*C* and D; *p* < 0.001). Conversely, this interaction is largely attenuated in its counterpart chemoresistant CECA cells (C13*) exhibited minimal mitochondrial Phb1-p-p53 (ser15) interaction (Fig. 6C, D; *p* < 0.05). Collectively, these results support the hypothesis that Phb1 is required for P-p53 (ser15)- Bak interaction that facilitates mitochondrial-mediated apoptosis, and is associated with CDDP-induced mitochondrial fission/fragmentation.

### Increased mitochondrial Phb1-p-p53(Ser15) interaction as a potential biomarker for chemosensitivity in OVCA

Based on previous in vitro findings and PLA study in human tumor sections that CDDP promotes the interaction of Phb1 and p-p53 (ser15) in mitochondria (Figs. 5, 6), we further examined whether stronger mitochondrial Phb1-p-p53 (ser15) interaction is also associated with better prognosis and chemo-responsiveness of OVCA patients. To this end, we performed PLA to examine the interaction between Phb1 and p-p53 (ser15) in advanced stages (III and IV) of high grade sub type of human ovarian tumor sections (Supplementary Table S3). Primary treatment OVCA cancer contains platinum containing agent [35]. Therefore, the length of progression free interval (PFI, time interval from the end of 1st chemotherapy to recurrence of cancer) [36, 37] is implemented as an indicator of chemoresponsiveness to Cisplatin or other platinum containing agent. Based on clinical oncology guideline, definition of length of 6 months (mo) is used as cut off time to differentiate between chemosensitivity (PFI > 6 mo), partially platinum-sensitive recurrence (6 mo ≤ PFI < 12 mo), and chemoresistance (PFI ≤ 6 mo), and totally platinum-sensitive recurrence (PFI ≥12 mo) [35]**.**

**Fig. 7:**
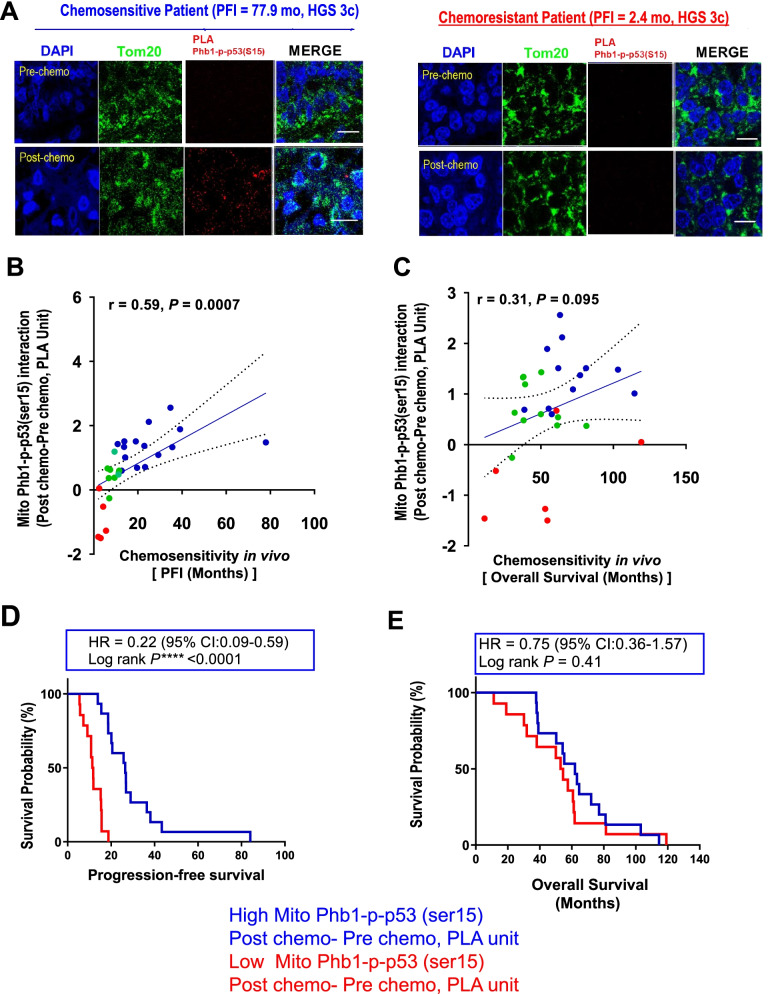
Chemotherapy-induced mitochondrial interaction of phb and p-p53 (Ser15) in vivo is associated with chemo-responsiveness in OVCA patients. **A** Images represent PLA signal from pre- and post- chemotherapy in OVCA IHC sections collected from chemosensitive patients (PFI = 77.9 month, mo) and chemoresistant patients (PFI = 2.4 mo), respectively. Scale bar: 10 μm. **B** & **C** Paired pre- and post- chemotherapy high-grade serous ovarian tumor sections from the same patients (*n* = 29, total 58 sections) were stained with anti-Phb1 and anti-p-p53 (ser15) antibodies. **B** progression free interval (PFI, from the end of 1st chemotherapy to recurrence of cancer), **(D)** Length of progression free survival (PFS, from starting of 1st treatment to recurrence of cancer),and **(C** & **E)** overall survival (OS, from starting of 1st treatment to last follow up) were measured based on its definition during the treatment. Number of Phb1-p-p53 (ser15) interactions (PLA unit: red spot) and its cellular localization [(Blue: DAPI) and (Green: TOM20)] were assessed. Correlation between increased PLA unit [(Phb1-p-p53(ser15)] and PFI (*n* = 29) **(B)** and OS (*n* = 29) **(C)** was analyzed [Increased mitochondrial PLA unit = Phb1-p-p53(ser15) interaction_(Post-chemotherapy)_ minus Phb1-p-p53(ser15) interaction_**(**Pre-chemotherapy**)**_]. Higher Phb1-p-p53 (ser15) interaction (increased mitochondrial PLA unit) is associated with better PFS **(D)** but not OS **(E)**, as determined by Kaplan – Meier analysis and hazard ratio (HR) determination. The correlation was analyzed using Pearson (r) method and Kaplan Meier curved were stratified according to the log rank *p* method

When comparing the mitochondrial Phb1-p-p53 (ser15) interaction in post-chemotherapy sections compared with pre-chemotherapy sections, we observed a significant increase in mitochondrial Phb1-p-p53(ser15) interaction in a chemosensitive patient with longer PFI (PFI = 77.9 mo), but not in a chemoresistant patient with shorter PFI (PFI = 2.4 mo) (Fig. 7A). An increased mitochondrial Phb1-p-p53 (ser15) interaction [Phb1-p-p53(ser15)_(post-chemotherapy)_ **–** Phb1-p-p53(ser15)_(pre-chemotherapy)_].

positively correlates with better chemosensitivity (Fig. 7B, *r* = 0.59, *p* = 0.0007), whereas mild correlation was still observed between this interaction and overall survival (OS) (Fig. 7C, *r* = 0.31, *p* = 0.095).

Finally, Kaplan-Meier analysis indicates that patients (*n* = 29 high grade serous) with a higher mitochondrial enrichment of Phb1-p-p53 (ser15) interaction after chemotherapy had significantly better progression free survival (PFS) (Fig. 7D, *p* < 0.0001, median survival length of 26.51 mo vs. 11.54 mo) bue not overall survival (OS) (Fig. 7E, *p* = 0.41, with median survival length of 61.96 mo vs. 53.76 mo) than patients with lower mitochondrial Phb1-p-p53 (ser15) interaction. Collectively, these data suggest that increased mitochondrial Phb1-p-p53 (ser15) interaction in post-chemotherapy tumor section could possibly function as a biomarker and is strongly associated with higher chemo-responsiveness and better survival outcome (PFS and OS) in HGS patients with OVCA.

## Discussion

Our previous study has shown that p53-regulated L-Opa1 processing by Oma1 is a determining factor of apoptosis and chemoresistance in CECA cells [14]. The present study provides a better understanding of how p53 induces Oma1 activation, leading to subsequent L-Opa1 processing and cell death. We have shown that, in response to CDDP, activated p53 [p-p53 (ser15)] in sensitive cells binds to Bak, an important step for Oma1 activation. We also showed for the first time that Phb1 is involved in the action of p-p53(ser15) by facilitating its binding to Bak and promoting mitochondrial fragmentation. Moreover, Akt confers CDDP resistance in part by inhibiting the binding of p-p53 (ser15) to Bak, thereby attenuating Oma1-mediated L-Opa1 processing.

Phb1 is an evolutionarily conserved protein that is mainly located in mitochondria [17]. Mitochondrial Phb1 is located in the inner mitochondrial membrane and interacts with Phb2 to stabilize the mitochondrial genome, modulate mitochondrial dynamics, and facilitates the activation of mitochondrial apoptotic pathway [34]. The role of Phb1 in apoptosis remains controversial [17, 38]. Chowdhury and colleagues reported that Phb1 acts as a pro-survival factor during apoptosis in rat granulosa cells [39]. Merkwerth et al. demonstrated that conditional deletion of Phb2, an isoform of Phb1, enhances Opa1 processing and results in aberrant cristae morphogenesis and decreased cell proliferation [17]. In contrast, Peng et al. showed that the overexpression of Phb inhibited cell proliferation and enhanced vitamin D-induced antiproliferative action in breast cancer cells [23]. Phb1 has also been shown to negatively regulate human and mouse liver cancer tumorigenesis through the downregulation of key oncogenes such as c-MYC [38]. On the other hand, Anderson et al. recently reported a novel role of Phb1 and showed that it mediates the stabilization of the major mitochondrial phospholipid cardiolipin and regulates stress response and cell death through Oma1 turnover [40]. Our results demonstrate that Phb1 content was increased in response to CDDP in chemosensitive cells, but not in chemoresistant cells. Phb1 knock-down attenuates CDDP-induced apoptosis and mitochondrial fragmentation in chemosensitive cells, suggesting that Phb1 is a pro-apoptotic protein. Whether these observed differences in the role of Phb (tumor suppressive versus oncogenic) are due to differences in *TP53* and *AKT* mutational status, heterogeneity of the cancer cells examined [41] and/or the involvement of different Phb isoforms, are not clear and await future investigations.

Although different intracellular localization of Phb1, including nucleus, plasma membrane and mitochondria, has been demonstrated, our current study emphasizes the role of mitochondrial Phb1 [42]. As a transmembrane adaptor, membrane Phb1 regulates the cell signaling for membrane transport. Nuclear Phb1 modulates transcriptional activity by interacting with transcription factors or indirectly interacting with chromatin remodeling factors [18]. In prostate cancer, nuclear Phb1 has been reported to regulate cell cycle progression targeted by androgen [43]. Within the nucleus, Phb1 has also been shown to co-localize with various transcription factors, including p53, Rb, E2F, AIF, c-myc, and c-fos [34, 44]. We also consistently observed p53 - Phb1 interaction in the nucleus upon CDDP treatment. It is possible that p53 is involved in increasing Phb1 expression as transcriptional level and promotes p53-dependent apoptosis, as CDDP treatment subsequently increased Phb1 protein content in chemosensitive cells (shown in our study). We also consistently observed the p53 and Phb1 interaction in the nucleus. Guan et al. also reported that Phb1 binds to the p53 induced gene 3 (PIG3) promoter motif (TGTCC) directly, promoting p53-dependent apoptosis [45].

Phosphorylation of p53 at ser15 and ser20 (p53 activation) followed by mitochondrial targeting is a determining factor of CDDP-induced apoptosis in CECA cells [3, 4]. We have previously reported that p-p53 (ser15, but not ser20) binds to Phb1 in chemosensitive but not chemoresistant CECA cells in response to CDDP [14]. Our current results showed that this interaction is also evident in chemosensitive but not chemoresistant OVCA cells, suggesting Phb1-p-p53 (ser15) interaction may be important for CDDP-induced apoptosis and CDDP sensitivity. It has been demonstrated that the ability of Phb1 to suppress tumor formation is associated with the increased p53-mediated apoptotic response, and Phb1 directly interacts with p53 in HCT116 cells and Jurkat T cells in colon mucosa [46]. The p53-Phb1 interaction has been reported in MCF-7 breast cancer cells, and p53 transcription activity and Bax transcription are lower in the absence of Phb1 [21, 38]. To our knowledge, our study is the first to report the involvement of Phb1 in p53-mediated regulation of mitochondrial dynamics.

The integrity of the outer mitochondrial membrane (OMM) is controlled by BCL-2 family members [24]. The pro-apoptotic BCL-2 family member, Bak or Bax, oligomerizes into proteolipid pores and increases OMM permeability, cytochrome c release, and apoptosis. Opa1-mediated cristae opening was reported as Bax/Bak and BH3 dependent, and it is required for apoptosis independent of Bak oligomerization [9, 13]. Since Oma1 activation and subsequent L-Opa1 processing are required for mitochondrial fragmentation and apoptosis, it is not surprising that the former is regulated by Bak activation and increased OMM permeability, as reported earlier [47, 48]. Pietsch et al. reported that p53 DNA binding domain can directly bind Bak and facilitate Bak oligomerization [49]. Moreover, the observation that p-p53 (ser15) plays a role in Bak activation and apoptosis by targeting mitochondria and binding to Bak [25] is consistent with our contention that increased mitochondrial p-p53 (ser15) and its binding to Bak in chemosensitive cells during CDDP-induced mitochondrial fragmentation and apoptosis. Our results also provide new mechanistic insight into the role of Phb1 in promoting the p-p53 (ser15) - Bak complex in CDDP-induced mitochondrial fragmentation and apoptosis in gynecologic cancer cells. However, our study does not exclude the possibility that p53 may regulate mitochondrial dynamics through the pro-apoptotic factor Bax, another pro-apoptotic BCL-2 family member. Whether and how Bax is involved in p53 regulated Oma1-mediated L-Opa1 processing remains unclear.

Akt activation or its over-expression is a major determinant of chemoresistance. Our previous publication has shown that Akt confers chemoresistance in gynecologic cancer cells by inhibiting p53 phosphorylation and mitochondrial translocation [3, 4]. Our current results show that inhibiting Akt in chemoresistant cells increased p-p53 (ser15) content and sensitized the cells to CDDP. These responses are associated with increased Oma1 cleavage (activation), L-Opa1 processing and mitochondrial fragmentation. We have therefore demonstrated for the first time that Akt confers chemoresistance at least partly via inhibiting p-p53 (ser15)-regulated Oma1-mediated L-Opa1 processing and mitochondrial fragmentation.

In the present studies and our previous report [14], we have described the dysregulation of mitochondrial dynamics in chemoresistant CECA and OVCA cells, which include: hyperfusion of mitochondrial morphology, and suppressed mitochondria fragmentation when challenged with CDDP [14]. We also demonstrated the dyregulated pathways in the regulation of mitochondrial dynamics in chemoresistant cells, including the absence of p-p53 (ser15) activation and cleaved short form of Oma1 (40 KDa), stabilized L-Opa1 (55KDa) and the absence of p-p53 (ser15)-Phb1-Bak interaction [14]. Whether and precisely how other proteins such as Drp1 and Mfn 1 and 2 contribute to chemoresistance in gynecologic cancer are not well understood and need to be further investigated. The relationship between dysregulation of mitochondrial dynamics and chemoresistance is intriguing. Cells with hyper-fused mitochondria may be more resistant to external cell stresses, including chemotherapy, possibly due to high efficiency of energy production in mitochondria [50]. However, how L-Opa1 and Oma1 are involved in the regulation of energy metabolism and cell survival remains unclear.

Our PLA assay indicates that CDDP induces the mitochondrial Phb1-p-p53 (ser15) interaction, which is compromised in chemoresistant cells. Defects in p53 may suppress its interaction with Phb1 and translocation to the mitochondria/nucleus, consequently leading to attenuated mitochondrial fragmentation. It is also possible that CDDP induces the formation of p-p53 (ser15)- Bak complex at the mitochondria, increases permeabilization of OMM, resulting in intrinsic apoptosis in OVCA cells [14]. Considering that higher than 70% of ovarian cancer are high grade serous subtype with high p53 mutation rate (> 90%), it is likely that suppression of p53 and subsequent defect of mitochondrial fragmentation were observed in most cases of ovarian cancer, associated with chemoresistance.

To our knowledge, the present study is the first report demonstrating the association of mitochondrial Phb1-p-p53(ser15) with chemosensitivity and clinical outcomes in OVCA. Mitochondrial Phb1-p-p53 (ser15) interaction could potentially be exploited as a biomarker of chemosensitivity in epithelial OVCA. However, this interaction are limited to late cases (stage III and IV) of patients treated adjuvant chemotherapy or neo adjuvant chemotherapy since strong signature of mitochondrial Phb1-p-p53 (ser15) interaction was not observed in naïve tumor sections or cancer cells not treated with CDDP. Since our clinical samples in this study were obtained at all late clinical stage, a prospective study with early stage samples for this marker would benefit early detection and better treatment prognosis. Based on our in vitro and in vivo (tumor sections) results, examining mitochondrial Phb1-p-p53(ser15) interaction following chemotherapy will enable one to predict the clinical outcome and chemoresponsiveness, and on decision on possible alternate treatment strategy.

**Fig. 8:**
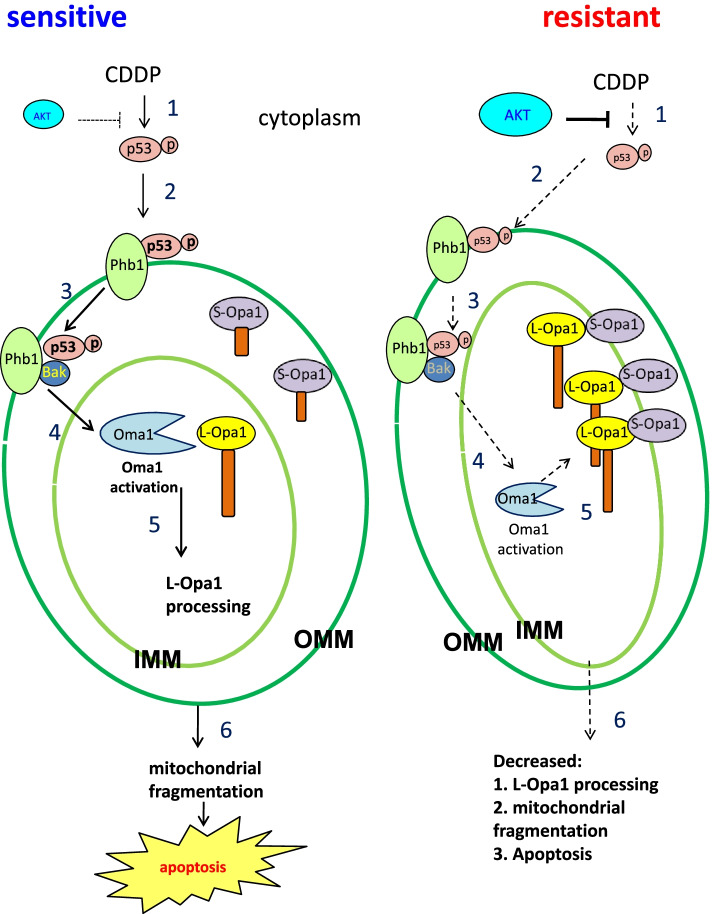
Hypothetical model illustrating in the regulation of mitochondrial fragmentation, apoptosis, and chemosensitivity in gynecologic cancer cells. In chemosensitive cells, 1) CDDP induces p53 phosphorylation (ser15), which accumulates in the mitochondria. 2) p-p53 (ser15) binds to Phb1. 3) The p-p53 (ser15)-Phb1 complex binds to Bak, inducing Bak activation. 4) The p-p53 (ser15)-Phb1-Bak complex activates Oma1 and 5) induces L-Opa1 processing to short form of Opa1. 6) This induces mitochondrial fragmentation in IMM, and subsequent apoptosis. In chemoresistant cells, 1) CDDP-induced p53 phosphorylation is minimal or absence, 2) suppressed stabilizing the Phb1-P-p53 (ser15) complex and 3) attenuatess the association of p-p53 (ser15)-Phb1-Bak complex. 4) It inhibits Oma1 activation in OMM. 5) L-Opa1 is protected from being processed in IMM, leading to the failure of CDDP to induce mitochondrial fragmentation and apoptosis. Solid arrow indicates activation and dashed arrow indicates suppressed activation. OMM and IMM indicate outer membrane inner membrane of mitochondria, respectively

We, for the first time, have demonstrated that the formation of p-p53 (ser15)-Bak-Phb1 complex in the mitochondria is necessary for Oma1-mediated L-Opa1 processing, mitochondrial fragmentation and changes in MOMP. Interestingly, MOMP may also activate Oma1 by stimulating self-cleavage [48], while Bak activation could cause MOMP and Oma1 activation as well as cytochrome c release [47]. Taken together, we propose that CDDP induces mitochondrial recruitment of p-p53 (ser15) uptake, and formation of p-p53 (ser15)--Phb1 complex, inducing MOMP and Oma1 self-cleavage, and subsequent Opa1 processing. In contrast, in chemoresistant cells, high Akt activity inhibits Oma1-mediated L-Opa1 processing by inhibiting p-p53 phosphorylation and mitochondrial targeting as proposed in hypothetical model (Fig. 8). Determining the molecular mechanisms by which p53 controls Oma1-mediated L-Opa1 processing may advance the current understanding of mitochondrial dynamics and apoptosis, and ultimately of the mechanisms of chemoresistance in human gynecologic cancer. Furthermore, identification of these mechanisms may support the possible application of mitochondrial Phb1-p-p53(ser15) interaction as a prominent signature for the identification of effective clinical intervention of OVCA to achieve better precision cancer medicine.

## Supplementary Information

**Additional file 1 Figure S1.** Protein-protein interaction analysis by PLA. Control of OVCA and CECA cell and description of Duo link PLA count method. **(A)** A2780s (ovarian cancer cells- OVCA), **(B)** C13 (cervical cancer cells – CECA), and **(C)** human ovarian tumour section without treatment of PLA reagents (contro. Blue represents DAPI (nucleus marker) and green represents TOM20 (mitochondria marker). **(D)** OV2008 cells treated without (CTL) or with CDDP were subjected to PLA assay. Using Duolink image tool, white dots (PLA signal) were counted either in mitochondria (green) or nucleus (Blue). Cell number was automatically assigned as shown by Duolink image tool. **(E)** PLA signal in each cell were counted, summed and averaged by number of cells as shown in table. **Figure S2.** CDDP increased Phb1 content and apoptosis in OV2008 cells, but not in C13* cells. **(A)** and **(B)** Comparison of Phb1 contents and apoptosis in OV2008 and C13* cultured with CDDP at different concentrations (**A**: 0–10 μM, 24 h) or for different duration (**B**: 0–24 h, 10 μM). Contents of Phb1 and GAPDH (loading control) were examined by Western blotting. Apoptosis was examined by Hoechst assay. Phb1 contents in OV2008 cells but not in C13* cells were significantly increased in the presence of CDDP in a concentration- (**A**; **p < 0.01 ***p < 0.001, *n* = 3) and time- (**B**; ***p < 0.001, *n* = 3) dependent manner. OV2008 cells exhibited higher apoptosis than C13* cells when treated with CDDP. Results are expressed as mean ± SEM (*n* = 3) and analyzed by 2-way ANOVA and Bonferroni post-hoc test. [**p < 0.01, ***p < 0.001, (versus CDDP = 0; ***A***) and (versus time = 0; **B**); *n* = 3]. **Figure S3**. p-p53 (ser15) interacts with Phb1 and Bak in response to CDDP in chemosensitive CECA cells, but not in chemoresistant cells. **(A)** OV2008 and C13* cells were treated with CDDP (0–10 μM, 6 h). Protein contents of Phb1, p-p53 (ser15), p-p53 (ser20), Bak and GAPDH were examined by Western blot. Protein-protein interaction was determined by IP-Western. **(B)** Cell lysates were immunoprecipitated with IgG (control; lanes 1) or Bak antibody. Bak immunoprecipitates were immunoblotted [IP: anti-Bak, WB: anti-Bak, −Phb1, −p-p53 (ser15 and ser20)]. Results show representative images from 3 independent experiments. Results are expressed as mean ± SEM (*n* = 3) and analyzed by 2-way ANOVA and Bonferroni post-hoc test. [**A** and **B**; **p < 0.01, ***p < 0.001 (compared to DMSO), *n* = 3]. **Figure S4.** Prolonged CDDP treatment induced mitochondrial localization of Phb1 in CECA cells. **(A)** OV2008 and **(B)** C13* CECA cells were cultured with CDDP (0, 10 μM, 0, 3, 6, and 24 h; DMSO as a vehicle), and were examined by confocal microscopy. Cellular localization of Phb1 (Red) and p-p53 (ser15) were shown in representative images. Green: Mitochondrial Marker and Blue: Nucleus marker (DAPI) Merge 1 indicates the merged image between Phb1 and p-p53 (ser15) whereas merge 2 indicates merged image between DAPI and Phb1.

**Additional file 2.**

## Abbreviations

CDDP: Cisplatin
L-Opa1: Long form of Opa1
Phb: Prohibitin

## References

[1] Siegel RL, Miller KD, Jemal A (2019). Cancer statistics, 2019. CA Cancer J Clin.

[2] Li J, Feng Q, Kim JM, Schneiderman D, Liston P, Li M, Vanderhyden B, Faught W, Fung MF, Senterman M (2001). Human ovarian cancer and cisplatin resistance: possible role of inhibitor of apoptosis proteins. Endocrinology.

[3] Yang X, Fraser M, Moll UM, Basak A, Tsang BK (2006). Akt-mediated cisplatin resistance in ovarian cancer: modulation of p53 action on caspase-dependent mitochondrial death pathway. Cancer Res.

[4] Fraser M, Bai T, Tsang BK (2008). Akt promotes cisplatin resistance in human ovarian cancer cells through inhibition of p53 phosphorylation and nuclear function. Int J Cancer.

[5] Han CY, Patten DA, Kim SI, Lim JJ, Chan DW, Siu MKY, Han Y, Carmona E, Parks RJ, Lee C (2021). Nuclear HKII-P-p53 (Ser15) interaction is a prognostic biomarker for Chemoresponsiveness and glycolytic regulation in epithelial ovarian Cancer. Cancers (Basel).

[6] Otera H, Ishihara N, Mihara K (2013). New insights into the function and regulation of mitochondrial fission. Biochim Biophys Acta.

[7] Shutt TE, McBride HM (2013). Staying cool in difficult times: mitochondrial dynamics, quality control and the stress response. Biochim Biophys Acta.

[8] Mishra P, Carelli V, Manfredi G, Chan DC (2014). Proteolytic cleavage of Opa1 stimulates mitochondrial inner membrane fusion and couples fusion to oxidative phosphorylation. Cell Metab.

[9] Patten DA, Wong J, Khacho M, Soubannier V, Mailloux RJ, Pilon-Larose K, MacLaurin JG, Park DS, McBride HM, Trinkle-Mulcahy L (2014). OPA1-dependent cristae modulation is essential for cellular adaptation to metabolic demand. EMBO J.

[10] Frezza C, Cipolat S, Martins de Brito O, Micaroni M, Beznoussenko GV, Rudka T, Bartoli D, Polishuck RS, Danial NN, De Strooper B, Scorrano L (2006). OPA1 controls apoptotic cristae remodeling independently from mitochondrial fusion. Cell.

[11] Mishra P, Chan DC (2016). Metabolic regulation of mitochondrial dynamics. J Cell Biol.

[12] Head B, Griparic L, Amiri M, Gandre-Babbe S, van der Bliek AM (2009). Inducible proteolytic inactivation of OPA1 mediated by the OMA1 protease in mammalian cells. J Cell Biol.

[13] Guillery O, Malka F, Landes T, Guillou E, Blackstone C, Lombes A, Belenguer P, Arnoult D, Rojo M (2008). Metalloprotease-mediated OPA1 processing is modulated by the mitochondrial membrane potential. Biol Cell.

[14] Kong B, Wang Q, Fung E, Xue K, Tsang BK (2014). p53 is required for Cisplatin-induced processing of the mitochondrial fusion protein L-Opa1 that is mediated by the mitochondrial Metallopeptidase Oma1 in gynecologic cancers. J Biol Chem.

[15] Farrand L, Byun S, Kim JY, Im-Aram A, Lee J, Lim S, Lee KW, Suh JY, Lee HJ, Tsang BK (2013). Piceatannol enhances cisplatin sensitivity in ovarian cancer via modulation of p53, X-linked inhibitor of apoptosis protein (XIAP), and mitochondrial fission. J Biol Chem.

[16] Merkwirth C, Martinelli P, Korwitz A, Morbin M, Bronneke HS, Jordan SD, Rugarli EI, Langer T (2012). Loss of prohibitin membrane scaffolds impairs mitochondrial architecture and leads to tau hyperphosphorylation and neurodegeneration. PLoS Genet.

[17] Merkwirth C, Dargazanli S, Tatsuta T, Geimer S, Lower B, Wunderlich FT, von Kleist-Retzow JC, Waisman A, Westermann B, Langer T (2008). Prohibitins control cell proliferation and apoptosis by regulating OPA1-dependent cristae morphogenesis in mitochondria. Genes Dev.

[18] Toska E, Shandilya J, Goodfellow SJ, Medler KF, Roberts SG (2014). Prohibitin is required for transcriptional repression by the WT1-BASP1 complex. Oncogene.

[19] Merkwirth C, Langer T (2009). Prohibitin function within mitochondria: essential roles for cell proliferation and cristae morphogenesis. Biochim Biophys Acta.

[20] Osman C, Merkwirth C, Langer T (2009). Prohibitins and the functional compartmentalization of mitochondrial membranes. J Cell Sci.

[21] Chander H, Halpern M, Resnick-Silverman L, Manfredi JJ, Germain D (2010). Skp2B attenuates p53 function by inhibiting prohibitin. EMBO Rep.

[22] Liu YH, Peck K, Lin JY (2012). Involvement of prohibitin upregulation in abrin-triggered apoptosis. Evid Based Complement Alternat Med.

[23] Peng X, Mehta R, Wang S, Chellappan S, Mehta RG (2006). Prohibitin is a novel target gene of vitamin D involved in its antiproliferative action in breast cancer cells. Cancer Res.

[24] Bender T, Martinou JC (2013). Where killers meet--permeabilization of the outer mitochondrial membrane during apoptosis. Cold Spring Harb Perspect Biol.

[25] Nieminen AI, Eskelinen VM, Haikala HM, Tervonen TA, Yan Y, Partanen JI, Klefstrom J (2013). Myc-induced AMPK-phospho p53 pathway activates Bak to sensitize mitochondrial apoptosis. Proc Natl Acad Sci U S A.

[26] Bauer TM, Patel MR, Infante JR. Targeting PI3 kinase in cancer. Pharmacol Ther. 2015;146:53-60. 10.1016/j.pharmthera.2014.09.006 25240910

[27] Domcke S, Sinha R, Levine DA, Sander C, Schultz N (2013). Evaluating cell lines as tumour models by comparison of genomic profiles. Nat Commun.

[28] Woo MG, Xue K, Liu J, McBride H, Tsang BK (2012). Calpain-mediated processing of p53-associated parkin-like cytoplasmic protein (PARC) affects chemosensitivity of human ovarian cancer cells by promoting p53 subcellular trafficking. J Biol Chem.

[29] Yang X, Fraser M, Abedini MR, Bai T, Tsang BK (2008). Regulation of apoptosis-inducing factor-mediated, cisplatin-induced apoptosis by Akt. Br J Cancer.

[30] Soderberg O, Gullberg M, Jarvius M, Ridderstrale K, Leuchowius KJ, Jarvius J, Wester K, Hydbring P, Bahram F, Larsson LG, Landegren U (2006). Direct observation of individual endogenous protein complexes in situ by proximity ligation. Nat Methods.

[31] Tsuyoshi H, Wong VKW, Han Y, Orisaka M, Yoshida Y, Tsang BK (2017). Saikosaponin-d, a calcium mobilizing agent, sensitizes chemoresistant ovarian cancer cells to cisplatin-induced apoptosis by facilitating mitochondrial fission and G2/M arrest. Oncotarget.

[32] Al-Bahlani S, Fraser M, Wong AY, Sayan BS, Bergeron R, Melino G, Tsang BK (2011). P73 regulates cisplatin-induced apoptosis in ovarian cancer cells via a calcium/calpain-dependent mechanism. Oncogene.

[33] Qureshi R, Yildirim O, Gasser A, Basmadjian C, Zhao Q, Wilmet JP, Desaubry L, Nebigil CG (2015). FL3, a synthetic Flavagline and ligand of Prohibitins, protects Cardiomyocytes via STAT3 from doxorubicin toxicity. PLoS One.

[34] Yang J, Li B, He QY (2018). Significance of prohibitin domain family in tumorigenesis and its implication in cancer diagnosis and treatment. Cell Death Dis.

[35] Benedet JL, Bender H, Jones H, Ngan HY, Pecorelli S (2000). FIGO staging classifications and clinical practice guidelines in the management of gynecologic cancers. FIGO committee on gynecologic oncology. Int J Gynaecol Obstet.

[36] Covens A, Carey M, Bryson P, Verma S, Fung Kee Fung M, Johnston M (2002). Systematic review of first-line chemotherapy for newly diagnosed postoperative patients with stage II, III, or IV epithelial ovarian cancer. Gynecol Oncol.

[37] Rosen DG, Yang G, Liu G, Mercado-Uribe I, Chang B, Xiao XS, Zheng J, Xue FX, Liu J (2009). Ovarian cancer: pathology, biology, and disease models. Front Biosci (Landmark Ed).

[38] Fan W, Yang H, Liu T, Wang J, Li TW, Mavila N, Tang Y, Yang J, Peng H, Tu J (2017). Prohibitin 1 suppresses liver cancer tumorigenesis in mice and human hepatocellular and cholangiocarcinoma cells. Hepatology.

[39] Chowdhury I, Branch A, Olatinwo M, Thomas K, Matthews R, Thompson WE (2011). Prohibitin (PHB) acts as a potent survival factor against ceramide induced apoptosis in rat granulosa cells. Life Sci.

[40] Anderson CJ, Kahl A, Fruitman H, Qian L, Zhou P, Manfredi G (2020). Prohibitin levels regulate OMA1 activity and turnover in neurons. Cell Death Differ..

[41] Jupe ER, Liu XT, Kiehlbauch JL, McClung JK, Dell’Orco RT (1996). Prohibitin in breast cancer cell lines: loss of antiproliferative activity is linked to 3′ untranslated region mutations. Cell Growth Differ.

[42] Peng YT, Chen P, Ouyang RY, Song L (2015). Multifaceted role of prohibitin in cell survival and apoptosis. Apoptosis.

[43] Gamble SC, Odontiadis M, Waxman J, Westbrook JA, Dunn MJ, Wait R, Lam EW, Bevan CL (2004). Androgens target prohibitin to regulate proliferation of prostate cancer cells. Oncogene.

[44] Fusaro G, Dasgupta P, Rastogi S, Joshi B, Chellappan S (2003). Prohibitin induces the transcriptional activity of p53 and is exported from the nucleus upon apoptotic signaling. J Biol Chem.

[45] Guan X, Liu Z, Wang L, Johnson DG, Wei Q (2014). Identification of prohibitin and prohibiton as novel factors binding to the p53 induced gene 3 (PIG3) promoter (TGYCC)(15) motif. Biochem Biophys Res Commun.

[46] Kathiria AS, Neumann WL, Rhees J, Hotchkiss E, Cheng Y, Genta RM, Meltzer SJ, Souza RF, Theiss AL (2012). Prohibitin attenuates colitis-associated tumorigenesis in mice by modulating p53 and STAT3 apoptotic responses. Cancer Res.

[47] Jiang X, Jiang H, Shen Z, Wang X (2014). Activation of mitochondrial protease OMA1 by Bax and Bak promotes cytochrome c release during apoptosis. Proc Natl Acad Sci U S A.

[48] Zhang K, Li H, Song Z (2014). Membrane depolarization activates the mitochondrial protease OMA1 by stimulating self-cleavage. EMBO Rep.

[49] Pietsch EC, Perchiniak E, Canutescu AA, Wang G, Dunbrack RL, Murphy ME (2008). Oligomerization of BAK by p53 utilizes conserved residues of the p53 DNA binding domain. J Biol Chem.

[50] Dhingra R, Kirshenbaum LA (2014). Regulation of mitochondrial dynamics and cell fate. Circ J.

