# Supplementary material for: Prohibitin 1 interacts with p53 in the regulation of mitochondrial dynamics and chemoresistance in gynecologic cancers
Source: J Ovarian Res. 2022 Jun 7;15:70. doi: 10.1186/s13048-022-00999-x (PMC9172162; doi:10.1186/s13048-022-00999-x)
Supplement: Supplementary file 1 — Additional file 1 Figure S1. Protein-protein interaction analysis by PLA. Control of OVCA and CECA cell and description of Duo link PLA count method. (A) A2780s (ovarian cancer cells- OVCA), (B) C13 (cervical cancer cells – CECA), and (C) human ovarian tumour section without treatment of PLA reagents (contro. Blue represents DAPI (nucleus marker) and green represents TOM20 (mitochondria marker). (D) OV2008 cells treated without (CTL) or with CDDP were subjected to PLA assay. Using Duolink image tool, white dots (PLA signal) were counted either in mitochondria (green) or nucleus (Blue). Cell number was automatically assigned as shown by Duolink image tool. (E) PLA signal in each cell were counted, summed and averaged by number of cells as shown in table. Figure S2. CDDP increased Phb1 content and apoptosis in OV2008 cells, but not in C13* cells. (A) and (B) Comparison of Phb1 contents and apoptosis in OV2008 and C13* cultured with CDDP at different concentrations (A: 0–10 μM, 24 h) or for different duration (B: 0–24 h, 10 μM). Contents of Phb1 and GAPDH (loading control) were examined by Western blotting. Apoptosis was examined by Hoechst assay. Phb1 contents in OV2008 cells but not in C13* cells were significantly increased in the presence of CDDP in a concentration- (A; **p < 0.01 ***p < 0.001, n = 3) and time- (B; ***p < 0.001, n = 3) dependent manner. OV2008 cells exhibited higher apoptosis than C13* cells when treated with CDDP. Results are expressed as mean ± SEM (n = 3) and analyzed by 2-way ANOVA and Bonferroni post-hoc test. [**p < 0.01, ***p < 0.001, (versus CDDP = 0; A) and (versus time = 0; B); n = 3]. Figure S3. p-p53 (ser15) interacts with Phb1 and Bak in response to CDDP in chemosensitive CECA cells, but not in chemoresistant cells. (A) OV2008 and C13* cells were treated with CDDP (0–10 μM, 6 h). Protein contents of Phb1, p-p53 (ser15), p-p53 (ser20), Bak and GAPDH were examined by Western blot. Protein-protein interaction was determined by IP-Western. [file 13048_2022_999_MOESM1_ESM.pdf]

SI Fig 1

A2780s Ctrl

B C13 Ctrl

C Human section Ctrl

A

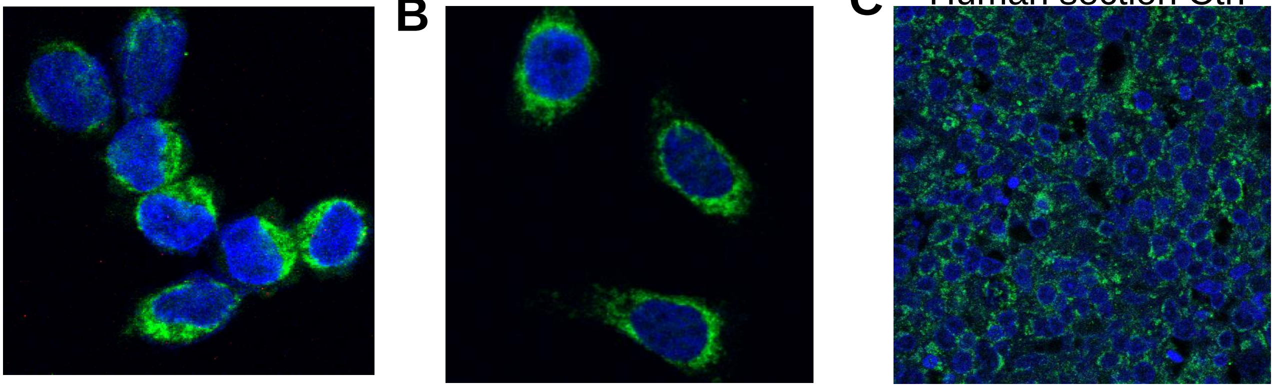

Green: Mitochondria
DAPI: Nucleus

D

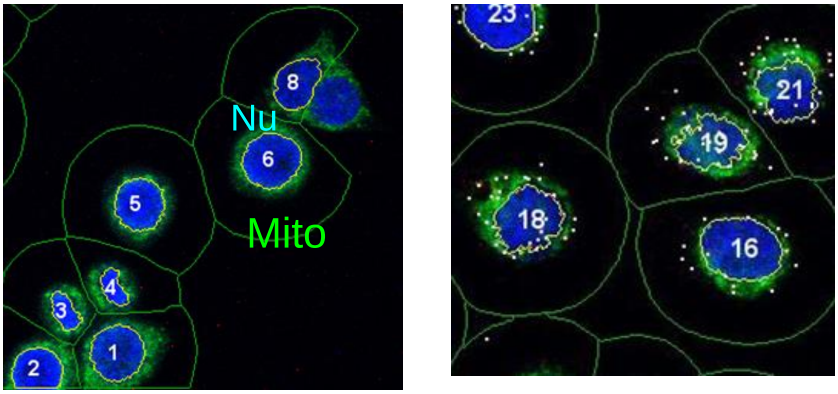

OV2008 CTL

OV2008 CDDP

E

Cell No.	OV2008CTL		Cell No.	OV2008+CDDP	
PLA	Mito	Nucleus	PLA	Mito	Nucleus
1	0	0	16	8	0
2	0	0	18	16	4
3	0	0	19	8	2
4	0	0	21	9	4
MEAN (PLA Sum/ # of cells)	0/4 (0)	0/4 (0)		42/4 (11)	10/4 (2.5)

Green: Mitochondria
DAPI: Nucleus
White dot: Counted PLA Signal

SI Figure 1 : Control of OVCA and CECA cell and description of Duo link PLA count method. **(A)** A2780s (ovarian cancer cells- OVCA), **(B)** C13 (cervical cancer cells – CECA), and **(C)** human sections without treatment of PLA reagents as control. Blue represents DAPI as nucleus marker and green represents TOM20 as mitochondrial marker. **(D)** OV2008 cells were not treated or treated with CDDP followed by treatment of PLA. Using Duolink image tool, white dots (PLA signa) were indicated and counted either in mitochondria (Tom20) or nucleus (DAPI: Blue). Cell number was automatically assigned as shown by Duolink image tool **(E)** PLA signal in each cell were counted and summed and were averaged by number of cells as shown in table.

SI Fig 2

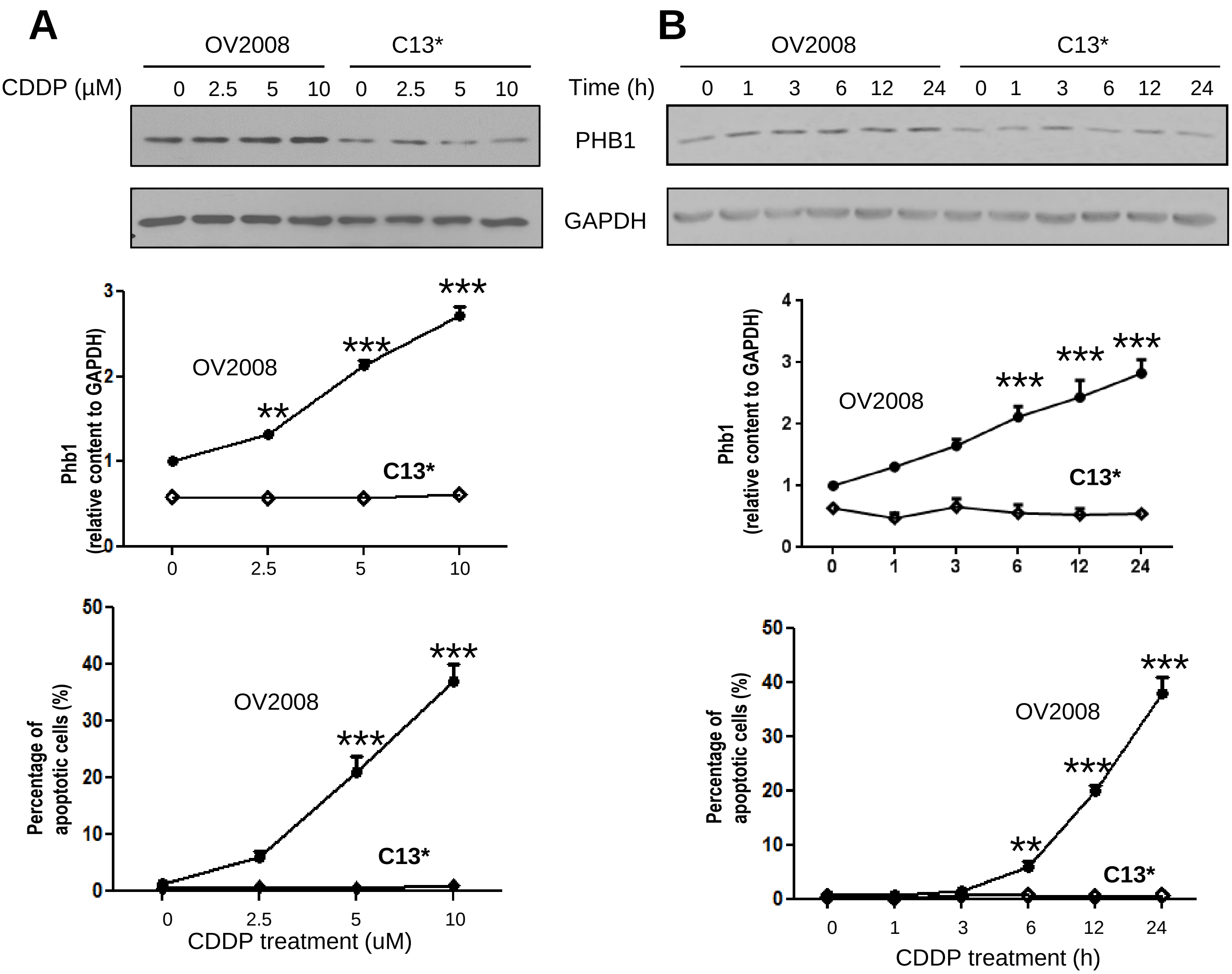

SI Figure 2 CDDP increased Phb1 content and apoptosis in OV2008 cells, but not in C13* cells. (***A***&***B***) Comparison of Phb1 contents and apoptosis in OV2008 and C13* cultured with CDDP at different concentrations (***A***: 0-10 µM, 24 h) or for different duration (***B***: 0-24 h, 10 µM). Contents of Phb1 and GAPDH (loading control) were examined by Western blotting. Phb1 contents in OV2008 cells but not in C13* cells significantly increased in the presence of CDDP in a concentration- (***A***; **p<0.01 ***p<0.001, n = 3) and time- (***B***; ***p<0.001, n = 3) dependent manner. Apoptosis was examined by Hoechst assay. OV2008 cells exhibited higher apoptosis than C13* cells when treated with CDDP (***A&B***; **p<0.01 ***p<0.001, n = 3).

SI Fig 3

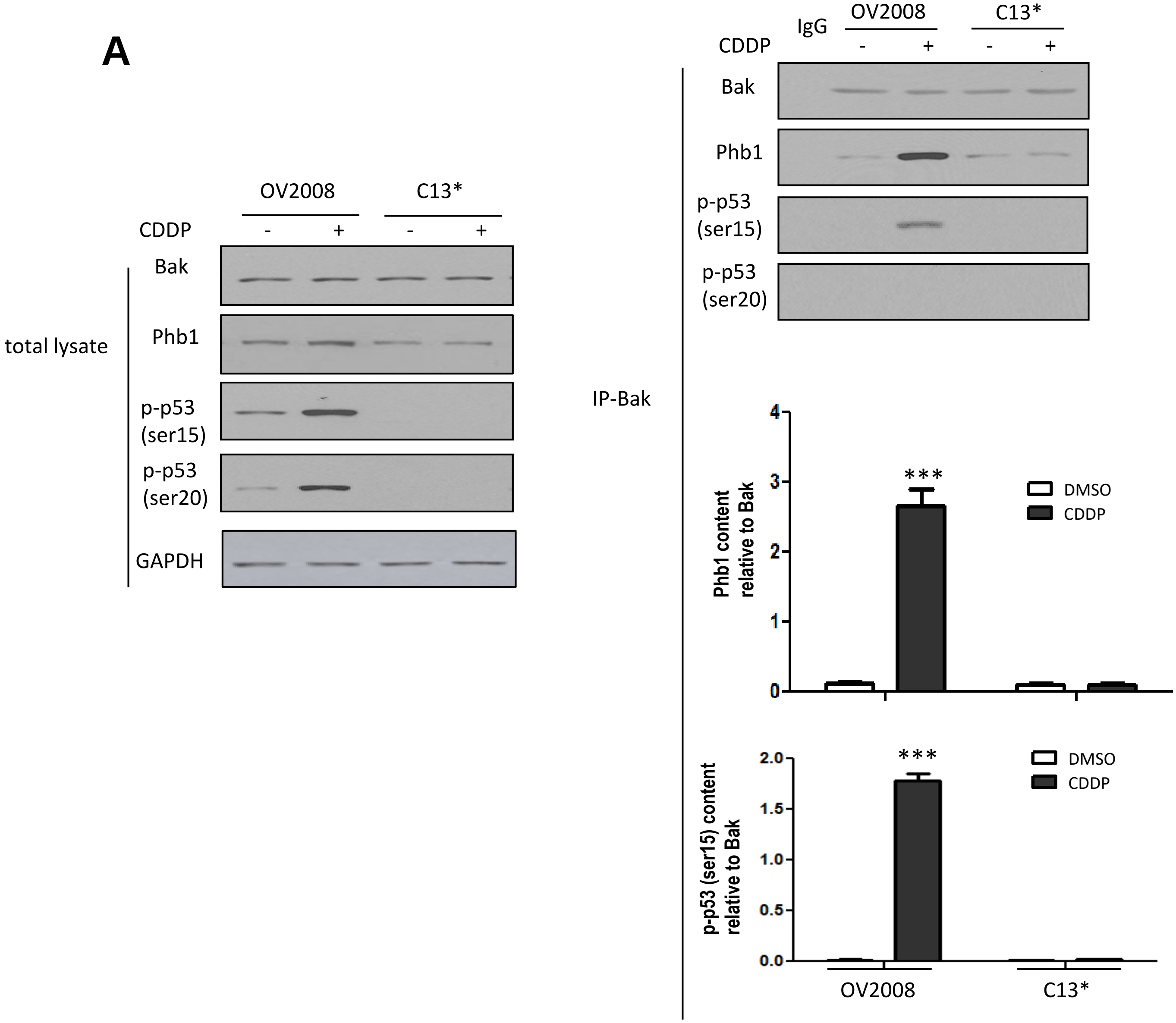

SI Fig 3. p-p53 (ser15) interacts with Phb1 and Bak in response to CDDP in chemosensitive CECA cells, but not in chemoresistant cells. OV2008 and C13* cells were treated with CDDP (0-10 µM, 6 h). Contents of Phb1, p-p53 (ser15), p-p53 (ser20), Bak and GAPDH were examined by Western blot. CDDP increased the content of both p-p53 (ser15 and ser20) in the chemosensitive cells but not in their resistant counterpart (n=3). Protein-protein interaction was determined by IP-Western. Cell lysates were immunoprecipitated with IgG (control; lanes 1) or Bak antibody. Bak immunoprecipitates were immunoblotted [IP: anti-Bak, WB: anti-Bak, Phb1, p-p53 (ser15 and ser20)]. CDDP increased the interaction of p-p53 (ser15, not ser20) -Bak (***$p<0.001$, versus DMSO, n=3) and Phb1-Bak (***$p<0.001$, versus DMSO, n=3) interactions in OV2008 cells but not in C13* cells. Results show representative images from 3 independent experiments.

SI Fig 4

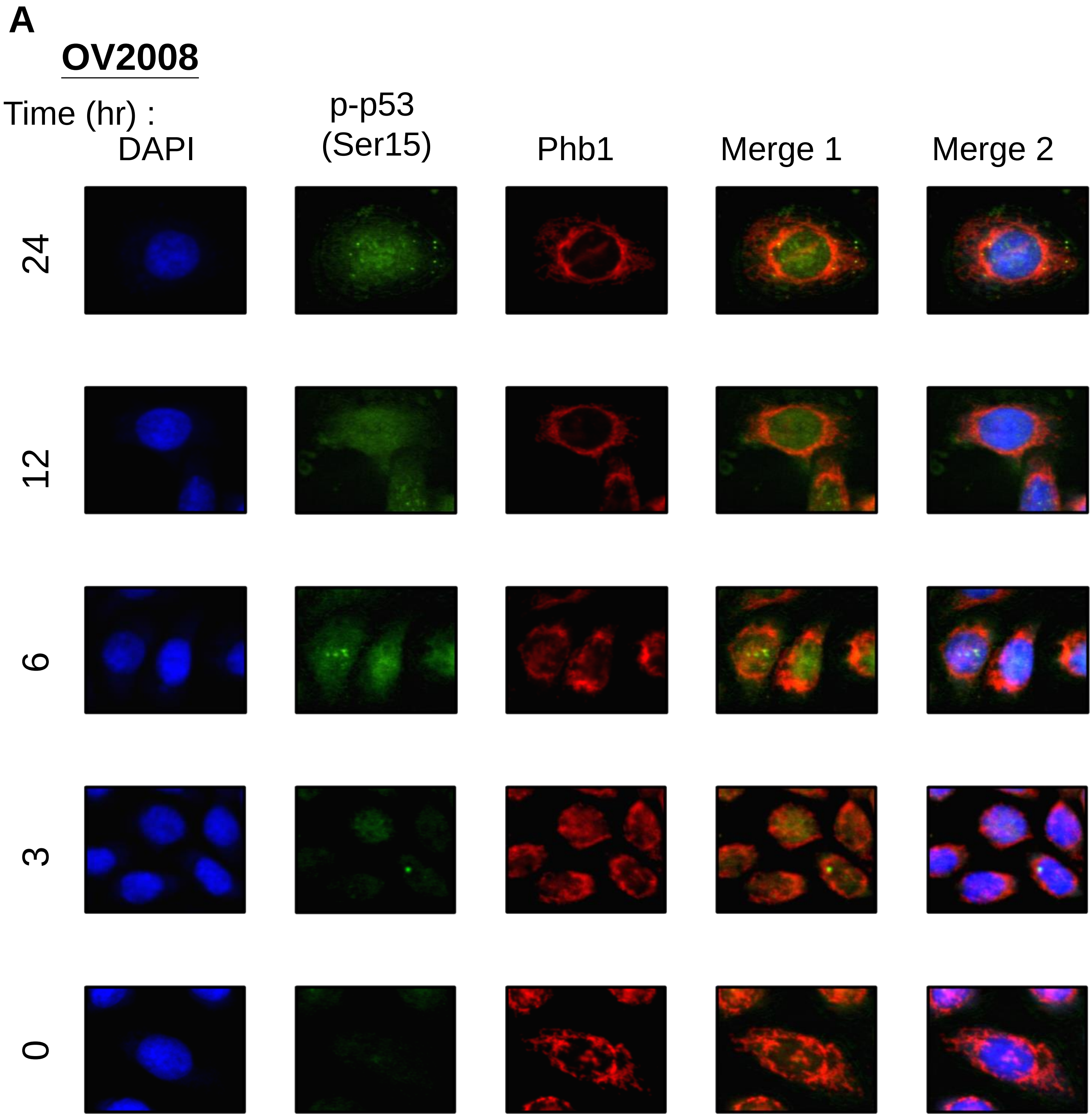

SI Fig 4 (Cont'd)

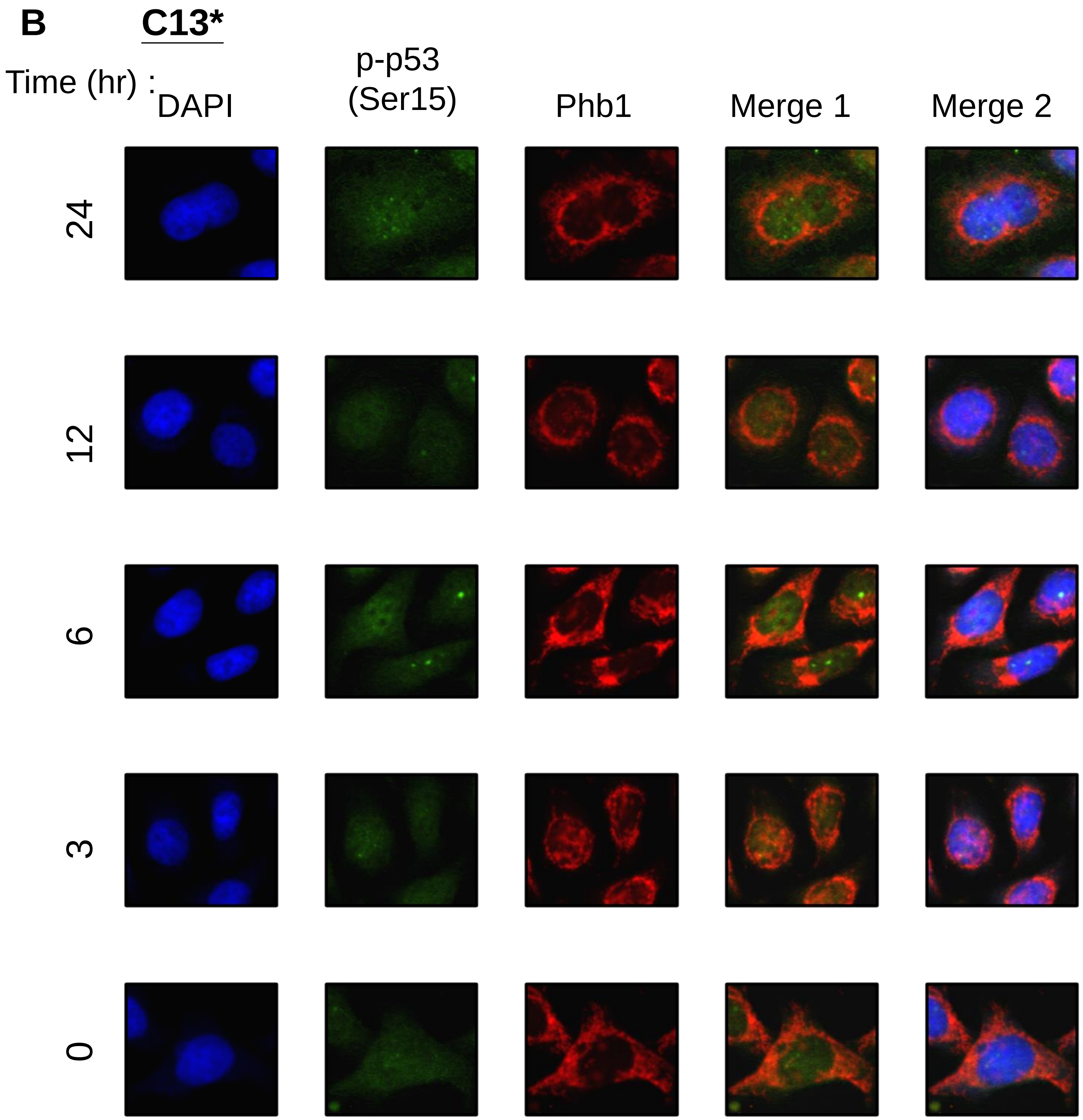

SI Figure 4. Prolonged CDDP treatment induced mitochondrial localization of Phb1 in CECA cells. (A) OV2008 and **(B)** C13* CECA cells were cultured with CDDP (0, 10 μM, 0, 3, 6, and 24 h; DMSO as a vehicle), and were subjected to confocal microscopy. Cellular localization of Phb1 (Red), p-p53 (ser15) (Green: Mitochondrial Marker), and DAPI (Blue: Nucleus marker) were shown in representative images. Merge I indicates the merged image between Phb1 and p-p53 (ser15) whereas merge 2 indicates merged image between DAPI and Phb1.
