# Supplementary material for: Prohibitin 1 interacts with p53 in the regulation of mitochondrial dynamics and chemoresistance in gynecologic cancers
Source: J Ovarian Res. 2022 Jun 7;15:70. doi: 10.1186/s13048-022-00999-x (PMC9172162; doi:10.1186/s13048-022-00999-x)
Supplement: Supplementary file 2 — Additional file 2. [file 13048_2022_999_MOESM2_ESM.docx]

**Supplementary Table S1. List of reagents**

**A. Primary antibodies**

| **Name** | **Reactive Species** | **Source** | **Dilution** | **Company** | **Catalogue No.** | **Application** |
| --- | --- | --- | --- | --- | --- | --- |
| Bak | H, Hamster, Pig, Mk | Rabbit pAb | 1/1000 (WB) | AbCam | Ab69404 | WB |
| HA tag | Independent | Mouse  mAb | 1/5000 (WB) | AbCam | Ab130275 | WB |
| Histone H3 | H, M, R, Mk | Rabbit  pAb | 1/5000 (WB) | Santa Cruz | SC-10809 | WB |
| Oma1 | H, M, R | Rabbit  pAb | 1/1000 (WB) | AbCam | Ab104316 | WB |
| Opa1 | H, M, R | Mouse  mAb | 1/1000 (WB) | BD Biosciences | 612607 | WB |
| Phb1 | H, M, R | Rabbit  pAb | 1/1000 (WB) | Santa Cruz | SC-377037 | WB, PLA |
| P-p53(Ser15) | H, M, R, Mk | Mouse  mAb | 1/5000 (WB) | Santa Cruz | SC-135772 | WB |
| P-p53(Ser15) | H, M, R, Mk | Mouse  mAb | 1/100 | Cell Signaling | 9286 | PLA |
| P-p53 (Ser20) | H, Mk | Mouse  pAb | 1/5000 (WB) | Santa Cruz | sc-18079 | WB |
| TOM20 | H, M, R | Mouse  mAb | 1/250 (IF)  1/5000 (WB) | Santa Cruz | sc-17764 | WB, IF |
| GAPDH | H, M, R | Rabbit  mAb | 1/10,000 | Ab cam | Ab181602 | WB |

**B. Secondary antibodies**

| **Host/Conjugate** | **Dilution** | **Company** | **Cat No.** | **Application** |
| --- | --- | --- | --- | --- |
| Goat Anti-Mouse Alexa Fluor 488 conjugate | 1/400 | Thermo  Fisher  Scientific | A11001 | IF |

**C. siRNA**

| **Name** | **Conc (nM)** | **Company** | **Catalogue** |
| --- | --- | --- | --- |
|  |  |  |  |
| Phb1 siRNA (h) | 0-100 nM | Origene | [SR303488](https://www.origene.com/catalog/rnai/sirna-oligo-duplexes/sr303488/prohibitin-phb-human-sirna-oligo-duplex-locus-id-5245) |
| Scramble siRNA (h) | 0-100 nM | Origene | SR30004 |

*Western Blotting (WB), ImmunoFluorescence (IF), Monoclonal Antibody (mAb), Polyclonal Antibody (pAb), H (Human), R (Rabbit), M (Mouse), Mk (Monkey), Cell Signaling Technology (Danvers, MA, USA), Santa Cruz Biotechnology (Santa Cruz, CA, USA), Abcam (Cambridge, MA, USA), LifeSpan BioSciences (Seattle, WA, USA), BD Biosciences (San Jose, CA, USA), Thermo Fisher Scientific (Waltham, MA, USA).

**Supplementary Table S2. Characterization of cell lines used in this study**

| **Cell line** | **Tumor origin** | ***TP53* status** | **Other Mutation** | **Chemosensitivity** |
| --- | --- | --- | --- | --- |
| A2780s | Ovarian endometrioid adenocarcinoma | Wild type | *PTEN/ARID1A* | Sensitive |
| A2780cp | Ovarian endometrioid adenocarcinoma | Mutant  V127F (Exon5)  R260S (Exon8) | *PTEN/ARID1A* | Resistant |
| OV2008 | Human papillomavirus-related cervical squamous cell carcinoma | Wild type | *PIK3CA; p.Glu545Lys (c.1633G>A*) | Sensitive |
| C13* | Human papillomavirus-related cervical squamous cell carcinoma | Null | *PIK3CA; p.Glu545Lys (c.1633G>A*) | Resistant |

**Supplementary Table S3. Information of patients in the study**

| **Characteristics**  **(patients, n=30)**  **paired sections (60)** | **Range** | **Patient studied population** | |
| --- | --- | --- | --- |
|  |  | **n/ Total** | **%** |
| **Age (years)** | < 50 | 3 / 29 | 10.3 |
|  | 50-60 | 14 / 29 | 48.3 |
|  | > 60 | 12/ 29 | 41.4 |
| **FIGO (Stage)** | III | 26 / 29 | 90 |
|  | IV | 3 / 29 | 10 |
| **Progression-free interval**  **(PFI)** | PFI > 12 m | 14 / 29 | 48.3 |
|  | 6 m < PFI ≤ 12 m | 10 / 29 | 34.5 |
|  | 6 m ≥ PFI | 5 / 29 | 17.2 |
| **Histologic sub-type** | High grade serous | 29 / 29 | 100 |

**Supplementary Table S3. Information of patients recruited in the study**

Table illustrates the clinical characteristics of the patients recruited for ovarian tumor immunohistochemistry section for PLA study. The majority of patients were older than 50 as menopause women. All subtypes of tumors were high-grade serous (100 %) and tumor samples were collected from patients with late stage as III (90%) or IV (10%). Half of patients had a recurrent disease and progression-free survival (PFS) of 12 months or more (48.3 %), while some patients had a recurrence between 6 to 12 months (34.5 %) and less than 6 months (17.2 %).
